# Protein–Protein Complex Stability Controls Substrate Scope in a *β*-Ketoacyl-ACP Reductase Specific for Medium Chains

**DOI:** 10.1002/anie.202508316

**Published:** 2025-09-01

**Authors:** Samuel J. Andrzejewski, Anika J. Friedman, Kathryn Mains, Annette Thompson, Nathaniel L. Hamel, Banumathi Sankaran, Peter H. Zwart, Michael R. Shirts, Jerome M. Fox

**Affiliations:** Department of Chemical and Biological Engineering, University of Colorado, Boulder, 3415 Colorado Avenue, Boulder, CO 80303, USA; Department of Chemical and Biological Engineering, University of Colorado, Boulder, 3415 Colorado Avenue, Boulder, CO 80303, USA; Department of Chemical and Biological Engineering, University of Colorado, Boulder, 3415 Colorado Avenue, Boulder, CO 80303, USA; Department of Chemical and Biological Engineering, University of Colorado, Boulder, 3415 Colorado Avenue, Boulder, CO 80303, USA; Department of Biochemistry, University of Colorado, Boulder, 3415 Colorado Avenue, Boulder, CO 80303, USA; Molecular Biophysics and Integrated Bioimaging, Lawrence Berkeley National Laboratory, 1 Cyclotron Road, Berkeley, CA 94720, USA; Molecular Biophysics and Integrated Bioimaging, Lawrence Berkeley National Laboratory, 1 Cyclotron Road, Berkeley, CA 94720, USA; Department of Chemical and Biological Engineering, University of Colorado, Boulder, 3415 Colorado Avenue, Boulder, CO 80303, USA; Department of Chemical and Biological Engineering, University of Colorado, Boulder, 3415 Colorado Avenue, Boulder, CO 80303, USA

**Keywords:** Assembly-line enzymes, Biocatalysis, Enzymology, Fatty acid synthesis, Molecular recognition

## Abstract

Assembly-line enzymes carry out multistep synthesis of important metabolites by using acyl carrier proteins (ACPs) to shuttle intermediates along defined sequences of active sites. Despite longstanding interest in reprogramming these systems for metabolic engineering and biosynthetic chemistry, the mechanisms underlying their reaction order remain poorly understood and difficult to control. Here we describe a *β*-ketoacyl-ACP reductase from *Pseudomonas putida* (*Pp*FabG4) with an unusual selectivity for medium chains and use it to explore the molecular basis of substrate specificity in enzymes that pull intermediates from fatty acid synthesis, a common route to specialized products. X-ray crystallography shows no obvious barriers to short-chain binding. Molecular simulations and supporting mutational analyses indicate that substrate preference arises instead from a weak enzyme–ACP interaction that is stabilized by medium acyl chains but not by short chains. Indeed, mutations that strengthen this interaction for *Pp*FabG4 or weaken it for *Ec*FabG, an *Escherichia coli β*-ketoacyl-ACP reductase with a broad substrate specificity, can enhance or reduce activity on short-chain substrates by over 100-fold. Our findings show how the stability of enzyme-ACP interactions can control substrate scope in promiscuous enzymes and guide the exchange of intermediates between (and within) assembly-line systems.

## Introduction

Assembly-line enzymes build molecules by using acyl carrier proteins (ACPs) to guide substrates along defined sequences of active sites.^[1–3]^ In fatty acid synthases (FASs), ACPs enable the iterative elongation of acyl chains required for lipid synthesis, energy storage, and signaling.^[4]^ Polyketide synthases (PKSs) exploit them for the ordered assembly of structurally diverse natural products.^[5,6]^ Both systems can exist as either multienzyme polypeptide chains (type I) or collections of discrete enzymes (type II).^[7–9]^ A detailed understanding of the mechanisms by which their active sites find ACPs with compatible cargo is essential for predicting and engineering their product profiles.

Structural studies of FASs and PKSs have begun to unravel the molecular basis of substrate channeling by ACPs. The type I PKS 6-deoxyerythronolide B synthase (DEBS) provides a framework. DEBS is a homodimer of three multifunctional polypeptides that together contain an N-terminal loading module, six elongation modules, and a C-terminal thioesterase. Each elongation module has one ACP and multiple enzymes.^[10]^ Structures obtained through cryogenic electron microscopy indicate that the ordered progression of substrates is maintained by i) differences in ACP binding affinity between proximal enzymes and ii) coordinated changes in protein conformation between dimeric subunits.^[6,11]^ Biochemical data suggest that the ACP preceding each module binds to the enzymes within it more tightly than its own “module-specific” ACP^[11,12]^—an example of the biophysical constraints required to balance inter- and intra-modular communication. The elongating ketosynthases (KSs) of the FAS of *Escherichia coli* exhibit similar coordination.^[13–15]^ These type II enzymes catalyze carbon–carbon bond formation via a ping-pong mechanism: The KS binds first to acyl-ACP, which transfers an acyl chain from its phosphopantetheine (Ppant) arm to the active site cysteine of the KS, and second to malonyl-ACP, which displaces the leftover holo-ACP before undergoing condensation with the acyl-enzyme to produce *β*-ketoacyl-ACP.^[7]^ X-ray crystallography, molecular simulations, and mutational analyses suggest that two flexible loops, which separate the active site and bound ACP, maintain reaction order through an allosteric relay system.^[15]^ Altogether, detailed biophysical analyses of ACP-enzyme interactions have uncovered important mechanisms for maintaining reaction order, but strategies for altering this order—or for predicting substrate preference—remain largely undeveloped.^[16–18]^

The FAS of *E. coli* is a valuable model for studying molecular recognition by assembly-line enzymes (Figure S1). This nine-enzyme type II system builds fatty acids in three steps^[19]^: i) initiation, the production of *β*-ketobutyryl-ACP from acetyl-CoA and malonyl-ACP, ii) elongation, the iterative reduction of *β*-ketobutyryl-ACP and condensation with malonyl-ACP (a two-carbon addition), and iii) termination, the removal of acyl-ACPs by acyltransferases for lipid assembly. In addition to supporting fatty acid synthesis, intermediate acyl-ACPs supply substrates for oleochemicals and polyketides.^[20–22]^ Prior work has shown that mutations in the acyl binding pockets of elongation and termination enzymes can shift their specificities toward short chains by obstructing the binding of longer ones^[23,24]^; however, the mechanisms by which enzymes draw off intermediate chain lengths to make specialized products remain unclear.

In this study, we used a fully reconstituted FAS from *E. coli* to characterize a novel oxidoreductase from *Pseudomonas putida* with an unusual selectivity for medium-chain substrates. Using X-ray crystallography, molecular modeling, and in vitro kinetic studies, we dissected the molecular basis of this preference. Our findings describe a new mechanism for controlling substrate specificity in ACP-mediated biocatalysis and shed light on the role of enzyme–ACP complex stability in guiding the progression of intermediates between (and within) assembly-line systems.

## Results and Discussion

**Figure 1.**
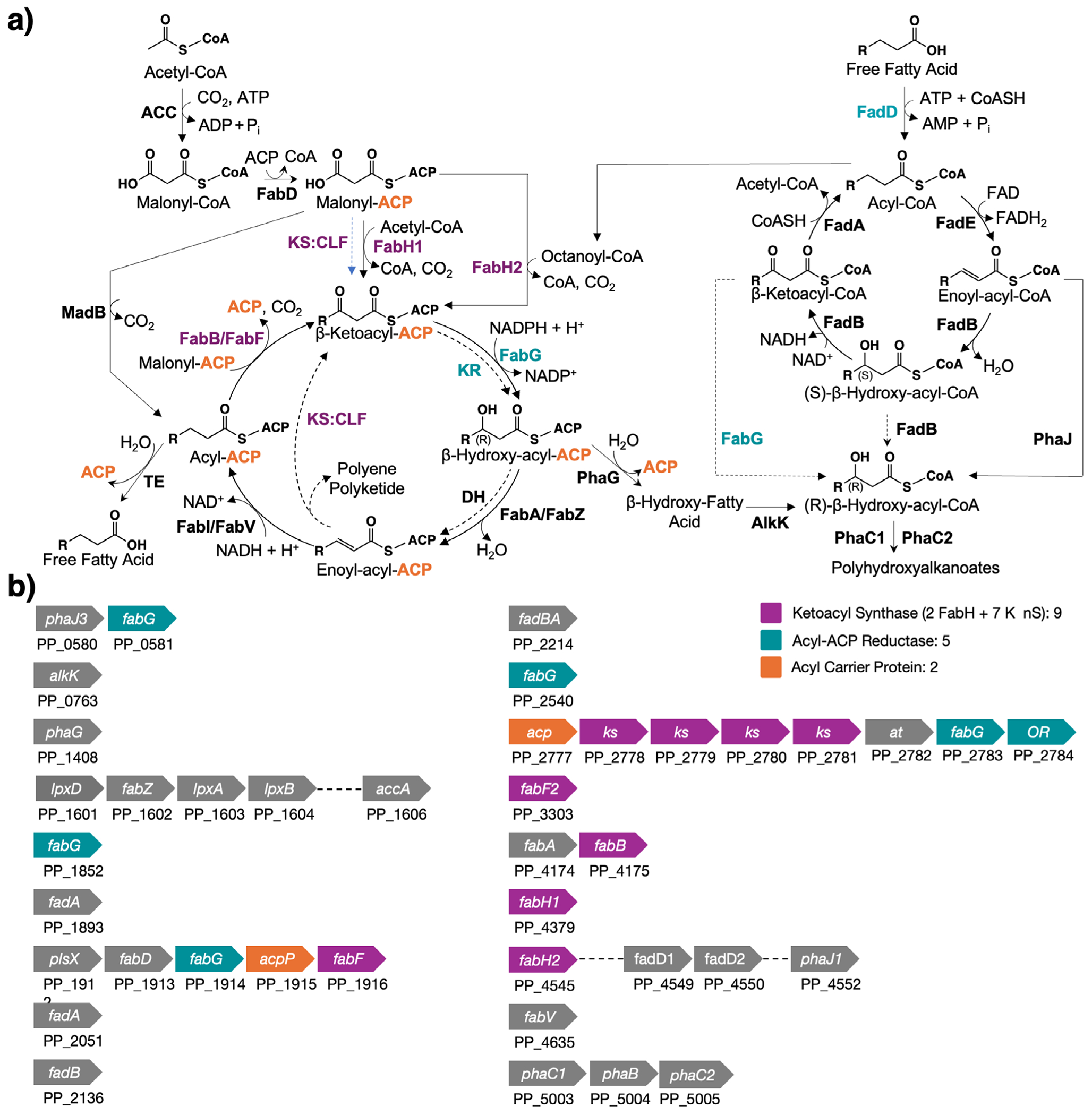
The fatty acid synthase (FAS) and related gene clusters in KT2440. a) Proposed pathways for fatty acid synthesis, *β*-oxidation, and polyhydroxyalkanoate production in KT2440 (solid arrows).^[36–39]^ Hypothetical related reactions catalyzed by auxiliary enzymes (dashed arrows) based on studies of bacterial type II FAS and PKS systems.^[40,41]^ b) Gene clusters identified by searching KEGG for homologues of the nine FAS proteins from *E. coli*. Highlights: enzymes examined in this study.

*Pseudomonas putida* KT2440 (KT2440) is a promising chassis for metabolic engineering. It can live on a wide range of carbon sources, thrives in diverse environmental conditions,^[25–28]^ and is industrially relevant,^[29,30]^ genetically tractable,^[31,32]^ and safe.^[33,34]^ Several groups have engineered this organism to produce valuable fatty acid derivatives (i.e., rhamnolipids^[35]^ and polyhydroxyalkanoates^[36]^), but gaps in understanding remain.^[37]^ In recent work, McNaught and colleagues identified three enzymes that can initiate fatty acid synthesis in KT2440 (Figure 1a)^[38]^: i) *Pp*FabH1, an orthologue of *E. coli* FabH (*Ec*FabH), condenses acetyl-CoA with malonyl-ACP to produce *β*-keto-butyryl-ACP; ii) *Pp*FabH2 acts on octanoyl-CoA to produce *β*-keto-decanoyl-ACP; and iii) *Pp*MadB decarboxylates malonyl-ACP to generate acetyl-ACP. The unusual selectivity of *Pp*FabH2 for medium chains implies a specialized sink (e.g., secondary pathway) for its products.

FAS-like enzymes are present in many important biocatalytic cascades (e.g., *β*-oxidation, polyhydroxyalkanoate metabolism, polyketide synthesis, nonribosomal peptide synthesis, and isoprenoid synthesis), a versatility that makes their functional roles difficult to ascertain from sequence data alone.^[40,42–44]^ To find enzymes that might draw off medium-chain acyl-ACPs, we searched the Kyoto Encyclopedia of Genes and Genomes (KEGG)^[45]^ for analogues of the nine FAS genes from *E. coli*. Our search produced nine KSs, six oxidoreductases—four *β*-ketoacyl-ACP reductases (KRs) and two putative enoyl-acyl-ACP reductases (ERs)—and two ACPs (Figure 1b). DeepFri, which uses graph convolutional networks to predict functions from structure and sequence data,^[46]^ predicted 8 genes to have functions consistent with KEGG, but 12 others, including *Pp*FabH1, had no assignments (Table S1). This inconsistency highlights the challenge of functional annotation for FASs.

One cluster of FAS-like genes (PP_2777-PP_2784) contained four sequential KSs, an organization suggestive of the heterodimeric KS/chain length factor complexes commonly found in PKSs.^[47]^ To probe its function further, we used Anti-Smash (Antibiotics and Secondary Metabolite Analysis Shell) 7.0, a bioinformatics tool designed to identify gene clusters involved in the production of secondary metabolites.^[48]^ To our surprise, this cluster showed a 100% match with a PKS from *Pseudomonas koreensis* known to produce koreenceine analogues; these antibacterial compounds are derived from medium-chain fatty acids and may help regulate the rhizosphere around plants (Figures S2 and S3).^[49,50]^ The koreenceine pathway, which is not annotated as a PKS of KT2440, is predicted to initiate synthesis with a 10-carbon substrate^[49]^; it is a candidate sink for medium-chain acyl-ACPs.

We evaluated the activity and substrate specificity of select FAS-like enzymes by using them to replace analogues in the *E. coli* FAS. In brief, we reconstituted this pathway with purified enzymes (i.e., *Ec*FabD, *Ec*FabH, *Ec*FabG, *Ec*FabA, *Ec*FabZ, *Ec*FabI, *Ec*FabB, *Ec*FabF, *Ec*ACP, and *Ec’*TesA, a promiscuous thioesterase with the signaling peptide removed; Figure S1) at concentrations that have been recapitulated in vivo trends in previous studies^[51–53]^ and measured FAS activity alongside several CoA precursors via NAD(P)H oxidation.

**Figure 2.**
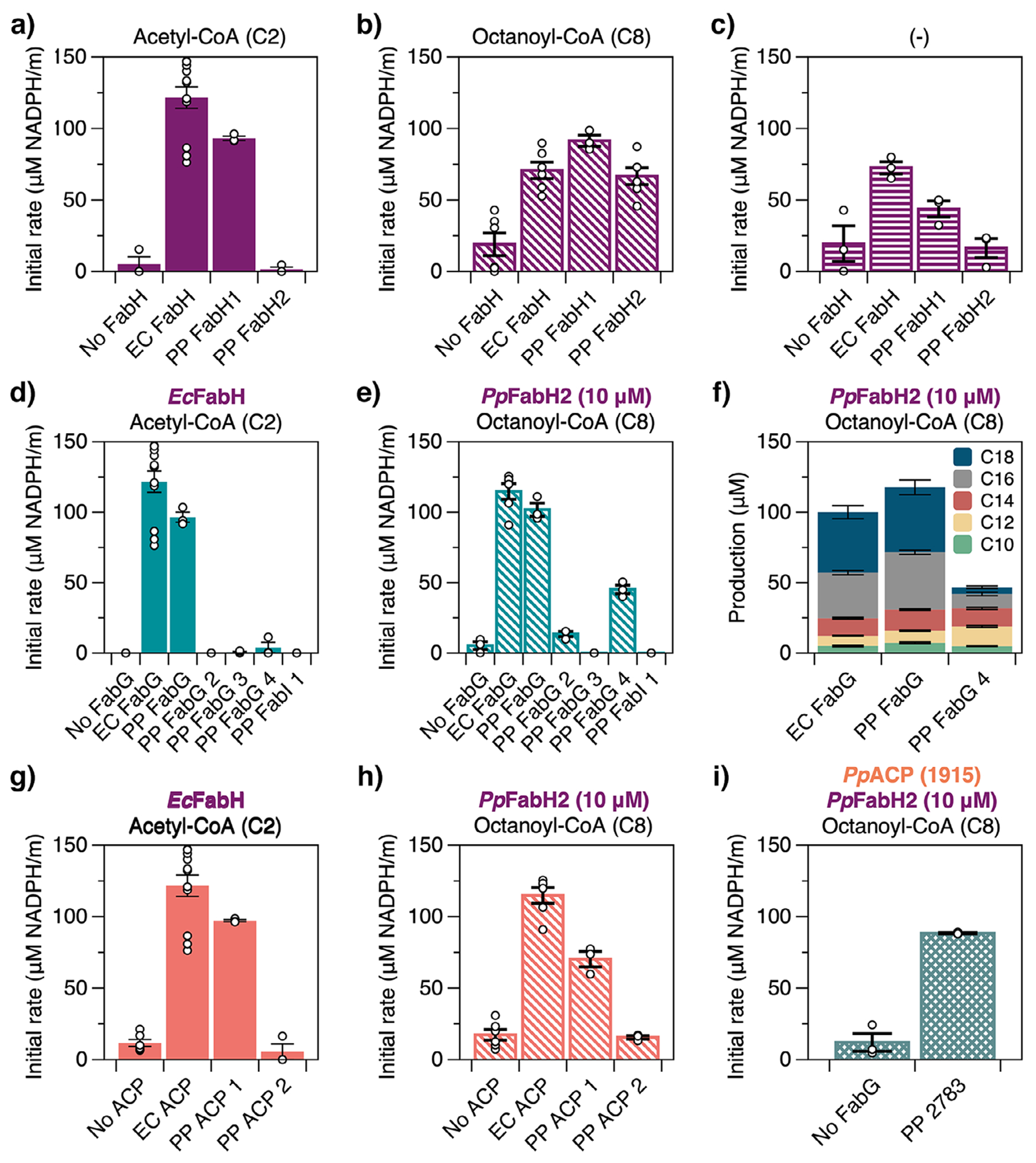
In vitro characterization of FAS-like enzymes from KT2440. Initial rates of fatty acid synthesis by reconstituted FASs with variants of a)–c) FabH, d), e), and i) FabG, or g) and h) ACP alongside various acyl-CoAs. f) Product profiles of the *E. coli* FAS with *Pp*FabH2 and variants of FabG. a)–i) All compositions include 1 μM of the indicated FabG or FabH variant (unless indicated otherwise), 10 μM ’TesA, 10 μM of holo-ACP, and 1 μM of all other FAS enzymes from *E. coli*. Initial rate reactions a)–e) and g)–i) include 1.3 mM NADPH, 0.5 mM malonyl-CoA, and 100 μM acyl-CoA, when included, while product profile reactions f) include 2.6 mM NADPH, 1.5 mM malonyl-CoA, and 300 μM acyl-CoA. Data were collected over 2.5 min (initial rate) or 120 min (product profile) and represent the mean and SE of *n* ≥ 3 technical replicates.

We began with PP_4379 (*Pp*FabH1) and PP_4545 (*Pp*FabH2): Consistent with prior work, only *Pp*FabH1 enabled FAS activity on acetyl-CoA (Figure 2a^[38]^). Both enzymes appeared active on octanoyl-CoA (Figure 2a,b), but the sustained activity of *Pp*FabH1 in the absence of either substrate (Figure 2c) suggests that this activity reflects the presence of acetyl-CoA generated by *Ec*FabB and *Ec*FabF. Kinetic models with this initiation route show a similar effect and confirm that errors in enzyme concentration cannot account for observed differences in FAS activity between reaction mixtures (Figure S4; SI Notes 2 and 3). We expanded our analysis of *Pp*FabH2 by evaluating additional substrates (Figure S5). Initial rate measurements show a narrow selectivity for C6 and C8 acyl-CoAs.

To identify a KR that might prefer the medium-chain acyl-ACPs generated by *Pp*FabH2, we tested each predicted KR from KT2440 in place of *Ec*FabG. Here, we used reconstituted FASs with either i) *Ec*FabH and acetyl-CoA or ii) *Pp*FabH2 and octanoyl-CoA. Our results show clear specialization: PP_1914 (*Pp*FabG1) enabled FAS activity on both substrates (Figures 2d,e and S6) and likely serves as the primary FabG of the KT2440 FAS. PP_2783 (*Pp*FabG4), however, conferred activity on only octanoyl-CoA, which suggests selectivity for *β*-keto-decanoyl-ACP. Further examination of FAS product profiles indicates that this enzyme is also active on longer chains (Figure 2f). *Pp*FabI1, which was predicted to be a KR or ER, had no measurable activity in place of *Ec*FabG or *Ec*FabI (Figure S6). The KRs that appeared inactive in our assays may require alternative substrates.

We concluded our survey of FAS-like proteins from KT2440 by testing the compatibility of its two ACPs with *Pp*FabH2 and *Pp*FabG4. PP_1915 (*Pp*ACP1), which shares 86% sequence identity with *Ec*ACP, permitted activity on octanoyl-CoA for both *Pp*FabH2 and *Pp*FabG4 (Figure 2h); however, PP_2777 (*Pp*ACP2), which has an unusual predicted structure (Figure S7), did not. The *Pp*ACP2 protein may be incompatible with the *E. coli* or the KT2440 enzymes under study or have an incorrect functional annotation. To conclude, our analyses suggest that *Pp*FabH2 generates *β*-keto-decanoyl-ACP, an intermediate drawn off by *Pp*FabG4 for koreenceine biosynthesis.

**Figure 3.**
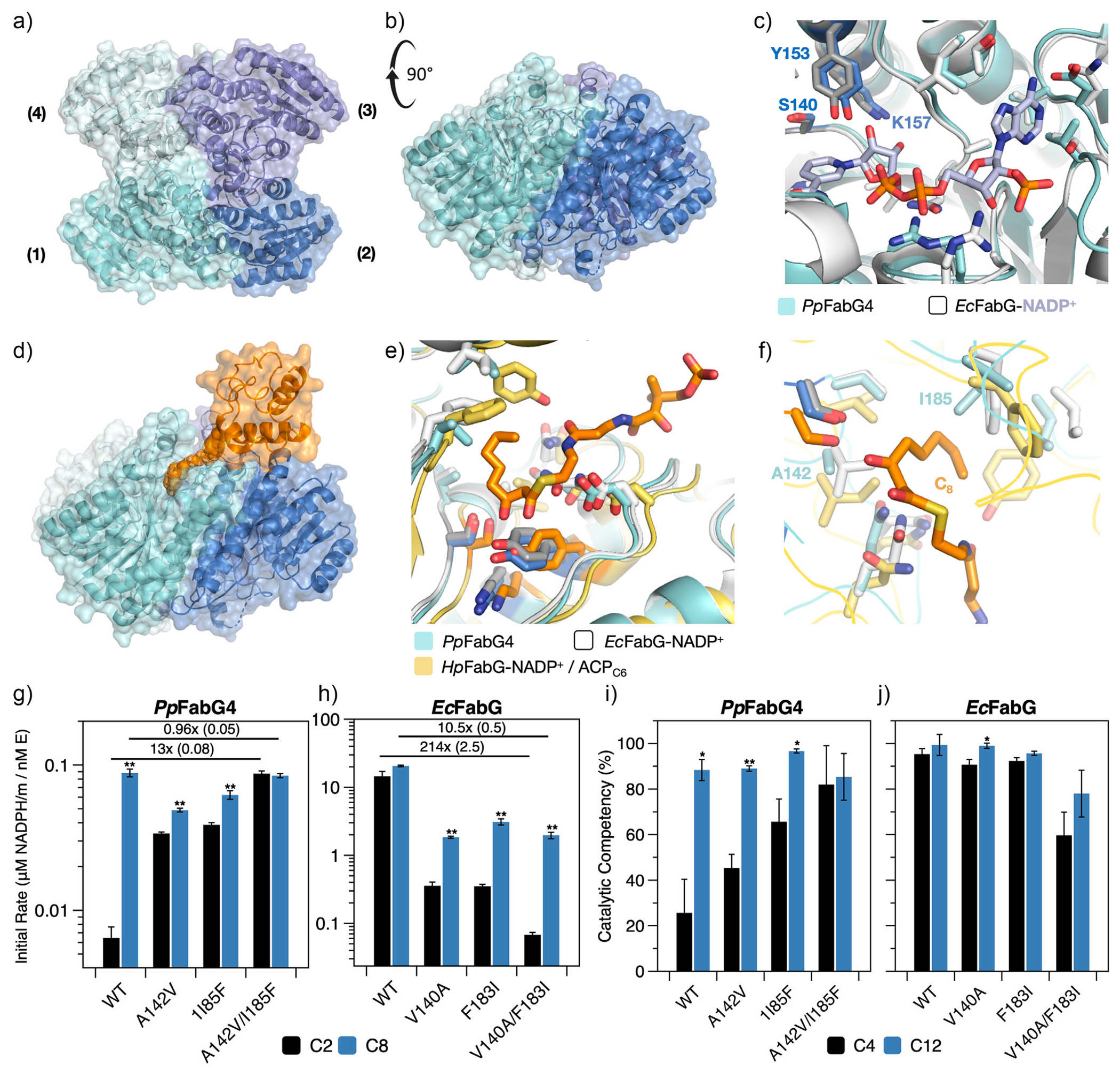
Crystal structure of the *β*-ketoacyl-ACP reductase, *Pp*FabG4, from KT2440. a) Top and b) side views of *Pp*FabG4 in its apo form (1.6 Å, PDB entry 9ng1). The enzyme forms a tetramer with D2 symmetry (i.e., a dimer of dimers A/D and B/C). c) Structural overlay of *Pp*FabG4 and *Ec*FabG-NADP^+^ (PDB entry 1q7b). The catalytic triad of both enzymes (highlighted and labeled for *Pp*FabG4) shows a competent conformation. d) Structural overlay of *Pp*FabG4 and *Hp*FabG-NADP^+^ bound to 3-beta-ketooctanoyl-ACP (C8-*β*kACP, PDB entry 8jfg) showing the tetramer of *Pp*FabG4 (blue surface) and the acyl-ACP (yellow surface with orange chain). e, f) Structural overlay of *Pp*FabG4, *Ec*FabG-NADP^+^, and *Hp*FabG-NADP^+^ bound to C8 *β*-ketoacyl-ACP showing e) the acyl binding pocket and f) two positions with shorter side chains in *Pp*FabG4. g) and h) Normalized initial rates of fatty acid synthesis by reconstituted *E. coli* FASs containing variants of g) *Ec*FabG and h) *Pp*FabG4 and (black) acetyl-CoA or (blue) octanoyl-CoA. FAS compositions: rate-limiting concentrations of FabG (Figure S14A,D and Table S4), 1 μM *Ec*FabH or 10 μM *Pp*FabH2, 10 μM ‘TesA, 10 μM holo-ACP, 1 μM of all other FAS enzymes, 1.3 mM NADPH, 0.5 mM malonyl-CoA, and 0.1 mM acyl-CoA. Measurement time: 2.5 min. i) and j) The catalytic competency of i) *Ec*FabG and j) *Pp*FabG4 bound to *β*-keto-acyl-ACPs with short (C4) and long chains (C12). Catalytic competency is defined by an enzyme-ACP complex with i) at least two interactions between FabG and ACP, ii) less than 3 Å between each donor hydrogen on S138 and Y151 and the *β*-carbonyl, and iii) less than 5 Å between the donor hydrogen on NADPH and the *β*-carbonyl. Data depicts the mean and SE of g) and h) *n* ≥ 3 technical replicates or i) and j) *n* = 3 simulations for each chain length. Independent *t*-tests between C2 and C8 activity for individual proteins: *, *p* < 0.05; **, *p* < 0.01.

Previous studies have used X-ray crystallography to study the apo and holo (i.e., cofactor) forms of *Ec*FabG, where NADPH binding reorganizes the catalytic triad (S138-Y151-K155) into a competent conformation^[54]^; though, this cofactor-induced conformational change is not shared by other FabG analogues.^[55]^ To study the unusual substrate specificity of *Pp*FabG4, we collected an X-ray crystal structure of the apo form. *Pp*FabG4 forms a tetramer similar to *Ec*FabG and other analogues (Figure 3a,b) and shows a catalytically competent conformation without cofactor (S140-Y153-K157, Figure 3c). As in previous FabG structures, the *Pp*FabG4 tetramer exhibits D2 symmetry—that is, a dimer of dimers (chains A/D and B/C). Sedimentation velocity analytical ultracentrifugation confirms that the tetramer is the dominant oligomeric state in solution (Figure S8 and SI Note 4), a finding consistent with prior work on other *β*-ketoacyl-ACP reductases.^[56]^

We used a structural overlay to search for residues that might disfavor the binding of short-chain substrates to *Pp*FabG4. Zhou et al. reported the first X-ray structure of a FabG-ACP complex in their study of FabG from *Helicobacter pylori* (*Hp*FabG;^[57]^); like *Ec*FabG, *Hp*FabG is active on both short and medium chains. Overlays of *Hp*FabG, *Ec*FabG, and *Pp*FabG4 suggest a shared binding mode (Figure 3d). The acyl chain from the *Hp*FabG-ACP complex sits in a nonpolar groove rich in aliphatic side chains (i.e., I143, I144, P185, G186, and F187 in *Hp*FabG; Figure 3e). For *Pp*FabG4, two side chains are smaller than those in *Hp*FabG and *Ec*FabG: Ala for Val or Ile (*Pp*FabG4_A142_/*Hp*FabG_I144_/*Ec*FabG_V140_) and Ile for Phe (*Pp*FabG4_I185_/*Hp*FabG_F187_/*Ec*FabG_F183_, Figure 3f). To assess the impact of these side chains on short-chain binding, we swapped them between *Pp*FabG4 and *Ec*FabG, and characterized mutant activity in reconstituted FASs containing C2 and C8 substrates—that is, with reaction mixtures that generate C4 and C10 *β*-ketoacyl-ACPs (*β*kACPs; Figure 3g). To our surprise, incorporating longer side chains into *Pp*FabG4 (A142V, I185F, and A142V/I185F) enhanced FAS activity on C2 by 13-fold and left activity on C8 nearly unaltered (Figure 3g), while swapping shorter side chains into *Ec*FabG (V140A, F183I, and V140A/F183I) reduced FAS activity on both chains, though much more so for C2 than for C8 (~210-fold vs. 10-fold, respectively, for double mutants relative to WT; Figure 3h). These findings suggest that expanding the acyl binding pocket can reduce activity on short-chain substrates, perhaps by removing stabilizing interactions.

**Figure 4.**
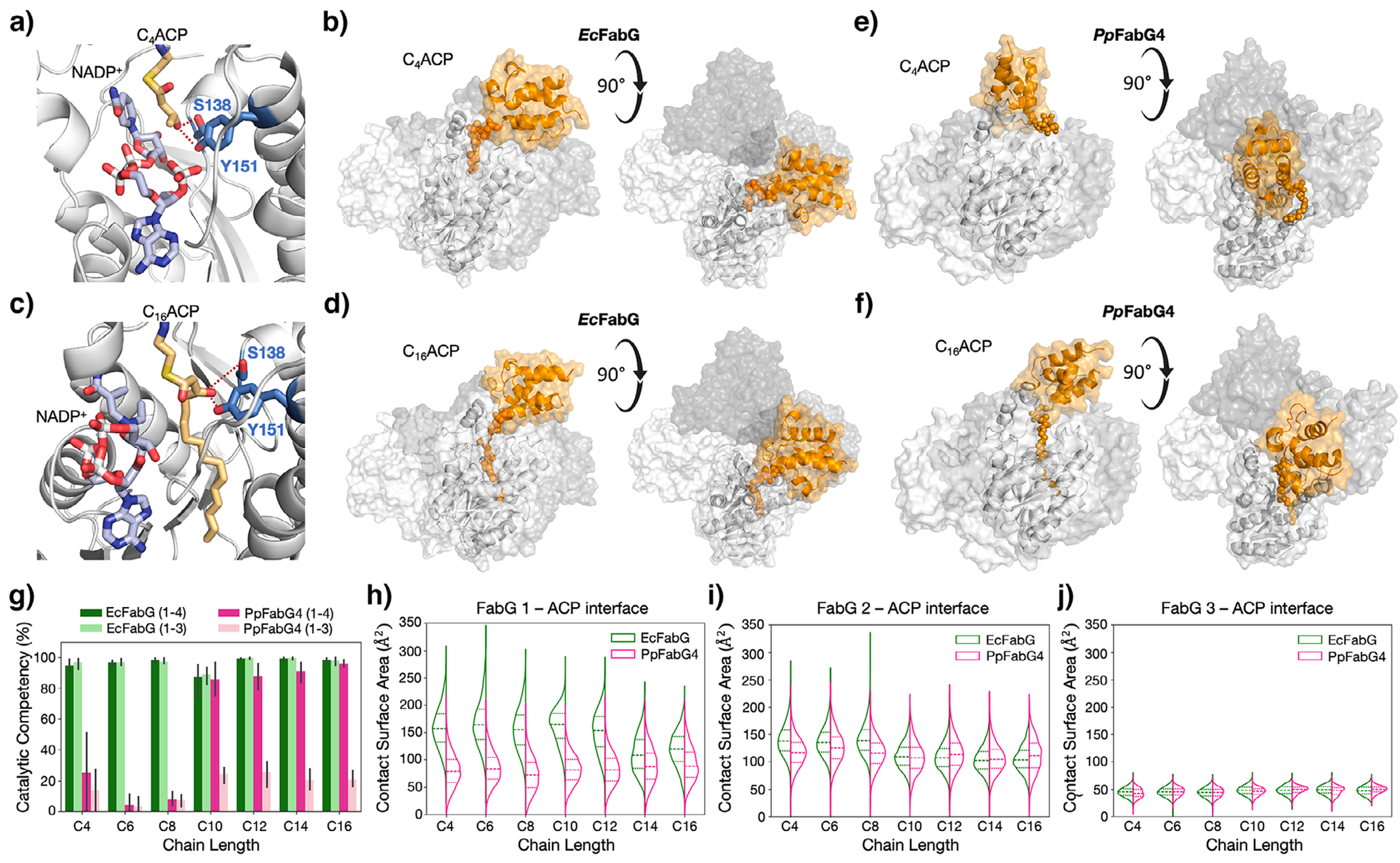
MD reveals differences in intermolecular contacts at the FabG-ACP interface. a)–d) Centroids from MD trajectories show *Ec*FabG bound to a) and b) C_4_ACP and c) and d) C_16_ACP. Acyl chains shorter than C10 are too short to access the acyl binding pocket. ACP shifts its position between short- and long-chain substrates but binds stably for all lengths. e) and f) Centroids from MD trajectories for *Pp*FabG4 bound to e) C_4_ACP and f) C_16_ACP. Short-chain substrates cannot bind stably; ACP shifts its position and acyl chains enter solution. Long-chain substrates bind as with *Ec*FabG. g) Differences in the stability of *Ec*FabG-ACP and *Pp*FabG4-ACP complexes over all simulations. Catalytic competency is as defined in Figure 3i,j. In MD simulations, *Ec*FabG is stable as a tetramer (1–4) and trimer (1–3) for all chain lengths, while *Pp*FabG4 is stable only as a tetramer bound to long-chain substrates. The trimeric form destabilizes at both FabG-ACP and inter-monomer interfaces early in simulations. h)–j) In simulations carried out with tetramers, we compared the contact area between ACP and each member of the tetramer, where numbering scheme started at the catalytic (1) subunit and increased counterclockwise. The fourth subunit, which does not form stable nonbonded interactions with ACP, is omitted. The total FabG-ACP contact interface is significantly decreased for *Pp*FabG4 compared to *Ec*FabG, particularly at the FabG(1)-ACP interface. For a)–f), we show the centroid from clustering on protein backbone RMSD of *n* = 3 simulations. For g) and j), we carried out *n* = 3 simulations of the indicated complex at 500 ns per simulation (i.e., the probability distribution is cumulative across all three simulations).

To examine the impact of chain length on the stability of enzyme–substrate complexes, we turned to molecular dynamics (MD) simulations. To begin, we used a computational workflow to identify binding sites for *Ec*ACP on *EcFabG* and *Pp*FabG4—a naïve approach to eliminate potential bias from the *Hp*FabG-ACP structure. We chose *Ec*ACP because we used it in our assays, and it is well studied and nearly identical to *Pp*ACP1 (86% sequence identity). We ran three independent replica simulations (500 ns) for each complex. For both enzymes, ACP binds to a trimeric interface that has two-fold symmetry on each side of the tetramer—i.e., four binding sites. Our analysis focuses on simulations of tetramers with one substrate bound (Figure 4a–f); simulations of trimers were stable for only *Ec*FabG, an indication of weaker intermolecular interactions within *Pp*FabG4 (Figures 4g and S9).

For *Ec*FabG, simulations showed two binding modes. For short-chain substrates (C4–C8), the acyl chain could not bind stably to the acyl pocket. Even when initiated there, it caused NADPH ejection (Figure S10) and adopted a solvent-exposed conformation (Figures 4a,b and S10, S11). Medium chains (≥C10) stayed in the acyl pocket, formed stable contacts with *β*1, *β*2, *α*2, and *α*4, and retained NADPH (Figures 4c,d and S12). Perhaps most importantly, for all chain lengths, ACP remained stably bound but shifted contact surface with *Ec*FabG between short and long chains, which exhibited reduced contact surface area (Figure 4b,d,h–j). Our simulations indicate that short-chain complexes with *Ec*FabG are less stable, but not sufficiently so to cause dissociation.

*Pp*FabG4 also showed two classes of behavior. For short substrates (C4–C8), the acyl chain could not bind stably to the acyl binding pocket but, unlike with *Ec*FabG, ACP dissociated from the protein (Figure 4e,g). By contrast, long-chain substrates remained stably bound with acyl chains in the pocket (Figure 4f,g). These results suggest that *Pp*FabG4 cannot form stable complexes with short-chain substrates.

Next, we used molecular simulations to explore the mutational effects observed in our assays. For EcFabG, simulations showed that residues V140 and F183 form direct nonbonded interactions with the Ppant linker and facilitate the formation of additional Ppant–protein interactions, all of which are absent in *Pp*FabG4 (Figure S13). When we introduced these amino acids into *Pp*FabG4 (A142V, I185F, and A142V/I185F), they increased the stability of short-chain substrates (Figure 3i). Here, our stability assessment focuses on catalytically competent conformations, which we define as the percent of an MD trajectory in which the enzyme-ACP complex has i) at least two interactions between FabG and ACP, ii) less than 3 Å between each donor hydrogen on both S138 and Y151 and the *β*-carbonyl, and iii) less than 5 Å between the donor hydrogen on NADPH and the *β*-carbonyl. When we swapped the corresponding mutations out of *Ec*FabG (V140A, F183I, and V140A/F183I), they reduced the stability of short-chain substrates—though, the effect was subtle, relative to the impact on *Pp*FabG4 (Figure 3j). These results indicate that stabilizing interactions with the Ppant arm can enhance FabG activity on short-chain substrates.

**Figure 5.**
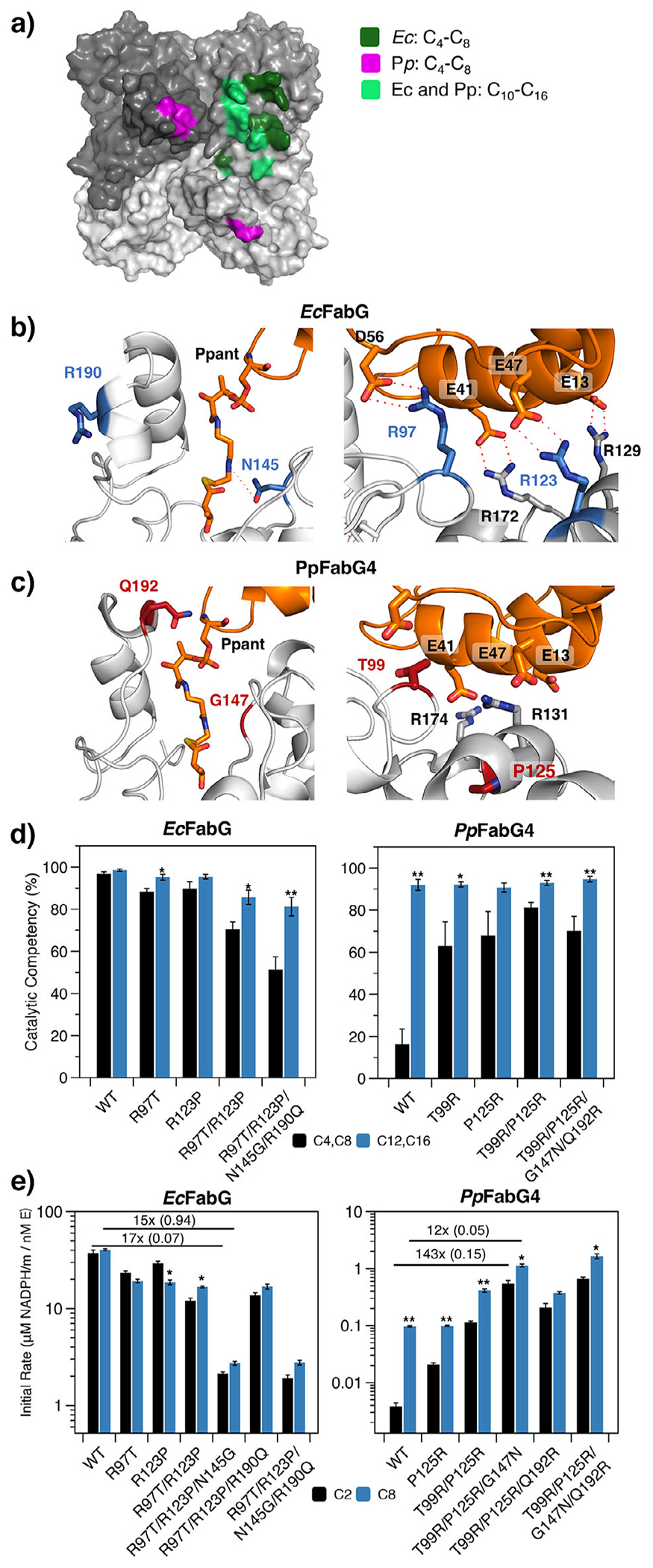
Mutations at the FabG-ACP interface can modulate substrate specificity. a) A surface representation of *Pp*FabG4 shows regions that make nonbonding interactions with ACP for (dark green) *Ec*FabG bound to C4–C8 (pink) *Pp*FabG4 bound to C4–C8, and (light green) both EcFabG and *Pp*FabG4 bound to C10-C16. b) and c) Centroids from MD trajectories show b) *Ec*FabG bound to C_4_ACP and c) *Pp*FabG4 with C_4_ACP overlaid from B to highlight lost interactions that disrupt stable binding. In *Pp*FabG4, T99 and P125 are incapable of forming salt bridges with the surface of ACP, and G145 cannot hydrogen bond to the Ppant arm; however, Q192 can stabilize ACP in a noncatalytically competent conformation (see Figure 4e). d) The catalytic competency (defined as in Figure 3i,j) of *Ec*FabG and *Pp*FabG4 bound to *β*-keto-acyl-ACPs with short (C4 and C8) and long chains (C12 and C16). Error bars correspond to standard error for *n* = 3 simulations for each chain length. Independent *t*-tests between C2 and C8 activity for individual proteins: *, *p* < 0.05; **, *p* < 0.01. e) Normalized initial rates of fatty acid synthesis by reconstituted *E. coli* FASs with variants of *Ec*FabG and *Pp*FabG4 with (black) acetyl-CoA or (blue) octanoyl-CoA. FAS compositions: rate-limiting concentrations of FabG (Figure S14E–L and Table S4), 1 μM *Ec*FabH or 10 μM *Pp*FabH2, 10 μM ‘TesA, 10 μM holo-ACP, 1 μM of all other FAS enzymes, 1.3 mM NADPH, 0.5 mM malonyl-CoA, and 0.1 mM acyl-CoA. Measurement time: 2.5 min. Data depicts the mean and SE of *n* ≥ 3 technical replicates. Independent *t*-tests between C2 and C8 activity for individual proteins: *, *p* < 0.05; **, *p* < 0.01.

Intrigued by the impact of stabilizing interactions on substrate specificity, we turned our attention to the FabG-ACP interface. Close inspection reveals critical differences between *Ec*FabG and *Pp*FabG4 (Figure S9). For both enzymes, ACP binds to a trimeric surface rich in nonpolar and positively charged residues that complement the nonpolar and negatively charged residues on ACP (Figures 5a–c and S15). When *Ec*FabG binds to short-chain *β*kACPs, it forms two unique interactions: R97 on the catalytic subunit and R123 on the neighboring unit form salt bridges with D56 and E47 on ACP, respectively (Figures 5b and S16). For *Pp*FabG4, the analogous residues—T99 and P125—cannot form salt bridges (Figures 5c and S16). When *Ec*FabG binds long-chain substrates, the FabG-ACP interface shifts to reduce the contact surface area with both the catalytic FabG (major) and an adjacent FabG (minor; Figures 4h,i and S16); stabilizing interactions with the acyl chains also increase (Figure S12A). The binding mode for long-chain substrates is nearly identical between *Ec*FabG and *Pp*FabG4, though the latter has fewer metastable nonbonded interactions (present for *<*50% of trajectories) and, thus, a smaller FabG-ACP binding interface (Figure 4g–i).

We probed the enzyme-ACP interface further by mutating key residues in *Ec*FabG and *Pp*FabG4. For each enzyme, we chose two sets of residues with the potential to influence substrate binding, denoted as *Ec*FabG (*Pp*FabG4): i) primary residues, which include R97 (T99) and R123 (P125), exhibited prominent differences between the FabG-ACP interfaces of *Ec*FabG and *Pp*FabG4 in our simulations, and ii) secondary residues, which include N145 (G147) and R190 (Q192), exhibited peripheral interactions that we speculated might affect overall binding affinity (Figure 5a–c). We began by swapping primary residues: R97T (T99R) and R123P (P125R) in *Ec*FabG (*Pp*FabG4). For *Ec*FabG, the primary mutations decreased the catalytic competency for short chains (C4 and C8) much more than long chains (C12 and C16; Figure 5d). For *Pp*FabG4, the complementary mutations increased the stability of short-chain complexes but had little effect on long chains. For secondary residues, which stabilize short-chain acyl-ACP complexes in *Ec*FabG (i.e., N145, which interacts with the Ppant arm in these complexes) or noncatalytically competent ACP binding to *Pp*FabG4 (i.e., Q192, which binds to ACP), swapping amino acids between proteins destabilized *Ec*FabG binding to short-chain substrates, relative to long, but had little effect on the substrate preference of *Pp*FabG4 (Figure 5d).

Next, we tested select mutants in vitro. The mutants exhibited a broad range of catalytic activities, so we adjusted concentrations to access a regime in which each was rate limiting (Figure S14). For *Ec*FabG, the R97T, R123P, and N145G mutations (alone or in combination) caused a similar reduction in activity on both C2 and C8 substrates, an indication that they destabilize ACP binding for all chain lengths; R190Q had no measurable effect (Figure 5e). Analogous mutations in *Pp*FabG4 exhibited complementary effects with a larger substrate dependence. The T99R, P125R, and G147N mutations increased activity on both C2 and C8, but this shift was much more pronounced for C2 (143-fold) than for C8 (12-fold); as before, Q192R had a negligible effect (Figure 5e). These shifts indicate that stabilizing the FabG-ACP interface can improve overall activity and broaden substrate scope by buffering out differences in interactions between FabG and acyl chains of different lengths. In general, mutants of *Ec*FabG were much more active than corresponding variants of *Pp*FabG4 (Figures 5e and S14). The more moderate effect of interfacial mutations on the overall activity of *Ec*FabG, relative to those located near the Ppant arm, suggests that they may be insufficiently destabilizing to alter the substrate specificity of this enzyme. In our final analysis, we combined all mutations from both regions of *Ec*FabG and *Pp*FabG4. Together, they caused directionally consistent changes in overall enzyme activity (i.e., *Ec*FabG activity decreased further, relative to the interfacial mutants, and *Pp*FabG4 activity increased less substantially, as one might expect from the modest active *Ppant* mutants), but effects on substrate specificity were not additive, suggesting that mutational effects are interdependent (Figure S17).

## Conclusion

The enzymes of biocatalytic assembly lines must find their substrates within the cellular milieu, a challenging feat when many molecules look alike. This study explores the molecular basis of substrate specificity in an enzyme that draws off fatty acid intermediates, a common route to specialized metabolites. Using in vitro complementation of the *E. coli* FAS, we showed that *Pp*FabG4 is a *β*-ketoacyl-ACP reductase with an unusual selectivity for medium chains. X-ray crystallography shows no obvious barriers to short-chain binding near the active site. Molecular simulations and supporting mutational analyses indicate that substrate preference arises instead from reduced FabG-ACP stability—the result of weak interactions between the enzyme and both the Ppant arm of ACP and ACP itself—that sensitizes FabG to stabilization by medium acyl chains. This finding is exciting because it shows how adjustments to enzyme-ACP stability can attenuate the substrate scope of promiscuous enzymes. Rational changes to this stability in assembly-line systems—most plausibly, from the enzyme side of the protein–protein interface—provide another knob for tuning product profiles. Prior work on type I FASs^[58]^ and PKS^[59]^ has shown that single mutations at domain–domain interfaces can alter substrate channeling within these systems. Our study provides a framework for tuning the strength of these interactions to sensitize them to minor, yet energetically influential differences between substrates.

Our analysis of *Pp*FabG4 suggests a rationale for the role of *Pp*FabH2 in KT2440. One might logically ask why a microbe would need multiple enzymes to initiate fatty acid synthesis, particularly one that specializes in the production of a medium-chain intermediate already formed during acyl chain elongation. The low activity of *Pp*FabG4 (i.e., 100-fold lower than *Ec*FabG) provides a potential justification: *Pp*FabH2 may help elevate the intracellular concentration of medium-chain acyl-ACPs beyond the steady state levels achieved during fatty acid synthesis—a necessary assist for FabG variants that exploit weaker binding to ACP to select for longer chains. Future efforts to evaluate the impact of *Pp*FabH2 deletion on polyketide production could test this hypothesis.

This work supports a control framework in which a dynamic interplay between enzyme concentration, acyl chain binding, and enzyme-ACP stability dictates overall FAS activity. A single ACP must bind to multiple enzymes, each with a distinct binding surface. For type 2 systems, differences in enzyme concentration can compensate for differences in enzyme-ACP binding affinity; in *E. coli*, we have used shifts in enzyme ratios to alter substrate partitioning between enzymes and increase product-specific titers by 125-fold.^[53]^ For type 1 systems, enzyme-specific conformational constraints, which can alter ACP accessibility, may play a similar role.^[60]^ For the acyl binding pocket, size can set an upper limit on substrate scope. Prior work,^[61]^ including our own study of KSs,^[ 24]^ shows that bulky residues can block the binding of longer chains. Reductions in enzyme-ACP stability provide a complementary mechanism to restrict the binding of short chains. Ultimately, the balance between these biophysical influences determines the overall structure of FASs and related pathways. Continued efforts to understand this balance are essential for the rational design of assembly-line systems.

## Supplementary Material

Supporting Information

Supporting Tables

Additional supporting information can be found online in the Supporting Information section

## Data Availability

The data is available within the paper and its Supporting Information files. The diffraction data generated in this study have been deposited in the PDB database (www.rcsb.org) under accession codes: 9NG1 (FabG). Source data are provided with this paper.
