# Supplementary material for: Protein–Protein Complex Stability Controls Substrate Scope in a β-Ketoacyl-ACP Reductase Specific for Medium Chains
Source: Angew Chem Int Ed Engl. Author manuscript; Available in PMC 2025 Oct 2. (PMC12490732; doi:10.1002/anie.202508316)
Supplement: Supporting Information [file NIHMS2111876-supplement-Supporting_Information.pdf]

Protein-protein interfacial stability controls substrate specificity in a β-ketoacyl-ACP reductase specific for medium chains

Samuel J. Andrzejewski[a±], Anika J. Friedman[a±], Kathryn Mains[a±], Annette Thompson[a], Nathaniel L. Hamel[b], Banumtahi Sankaran[c], Peter H. Zwart[[c]], Michael R. Shirts[a], and Jerome M. Fox[a*]

[a]Department of Chemical and Biological Engineering, University of Colorado, Boulder, 3415 Colorado Avenue, Boulder, CO, 80303
[b]Department of Biochemistry, University of Colorado, Boulder, 3415 Colorado Avenue, Boulder, CO, 80303
[c]Molecular Biophysics and Integrated Bioimaging, Lawrence Berkeley National Laboratory, Berkeley, CA, 94720
[±]Authors contributed equally to this work.

SI Methods S2
Notes S1-S4. . . . . S14
Figures S1-S18. . . . . S18
Table S1-S7 . . . . S41
SI References S55

SI METHODS

AntiSmash 7.0 Analysis. We searched for functionally annotated gene clusters similar to PP_2777-PP_2786 from KT2440 (NC_002947.4) by using AntiSmash 7.0 with the detection strictness set to "loose", which casts a broad net for both well- and poorly characterized clusters, and several extra features: KnownClusterBlast, ActiveSiteFinder, SubClusterBlast, RREFinder and TFBS analysis. The PP_2777-PP_2786 gene cluster showed a 100% match with a PKS from *P. koreensis.* In AntiSmash, the percent match is the percentage of genes within the closest known cluster with a significant BLAST hit to genes with the region of question.

Materials and Reagents. We used NEB® Stable Competent *E. coli* and BL21(DE3) *E. coli* cells (New England Biolabs) for cloning and protein expression, respectively. We purchased malonyl CoA, acetyl CoA, butyryl CoA (C4-CoA), hexanoyl CoA (C6-CoA), octanoyl CoA (C8-CoA), decanoyl CoA (C10-CoA), and dodecanoyl CoA (C12-CoA) as coenzyme A lithium salts from Millipore Sigma, and the cofactors NADPH (Nicotinamide adenine dinucleotide phosphate) and NADH (Nicotinamide adenine dinucleotide) from Cayman Chemical and Thermo Fisher Scientific, respectively. We obtained isopropyl β- d-1-thiogalactopyranoside (IPTG) from Thermo Fisher Scientific and arabinose from Acros Organics. We bought fatty acid standards from Acros Organics (decanoic acid, myristic acid and pentadecanoic acid), Alfa Aesar (dodecanoic acid), and Millipore Sigma (all others); antibiotics from Thermo Fisher Scientific (carbenicillin and kanamycin sulfate) and Millipore Sigma (chloramphenicol); and media components from Thermo Fisher Scientific (tryptone, yeast extract, and S.O.C media). We purchased all chromatography columns from Cytiva.

Plasmid construction. We amplified all *P. putida* KT2440 genes from genomic DNA extracted from *P. putida* KT2440 cultures via the DNeasy Blood and Tissue kit (Qiagen). For overexpression, we added individual genes with N-terminal polyhistidine tags to pET28a via restriction digest or Gibson assembly. Carrie Eckert provided wild-type KT2440. Table S3 reports all primers.

Protein Expression and Purification. We overexpressed all FAS enzymes in *E. coli* as described previously. In brief, we transformed chemically competent BL21(DE3) cells with individual plasmids, recovered the cells in S.O.C. (Super Optimal broth with Catabolite repression) media (2% tryptone, 0.5% yeast extract, 10 mM NaCl, 2.5 mM KCl, 10 mM $MgCl_2$, 10 mM $MgSO_4$, and 20 mM glucose) for an hour, plated them on Luria–Bertani (LB) agar plates supplemented with antibiotic (10 g/L tryptone, 5 g/L yeast extract, 10 g/L sodium chloride; 50 μg/mL carbenicillin or kanamycin), and grew the cells at 37 °C overnight. Using individual colonies, we inoculated 20 mL cultures of LB media containing antibiotic (50 μg/mL kanamycin) and incubated them at 37°C and 225 rpm until the cultures become cloudy (~5 hours). We diluted the 20-mL cloudy cultures into 1 L of rich induction media supplemented with antibiotics (20 g tryptone, 10 g yeast extract, 5 g sodium chloride, 0.4% glucose, 72 mL of 5X M9 solution; 50 μg/mL carbenicillin or kanamycin) and grew the 1-L cultures at 37°C and 225 rpm until OD_{600} reached 0.4-0.8. At this OD, which is indicative of mid-exponential growth, we added 0.5 mM IPTG and transferred the cultures to 22°C and 225 rpm for another 14-18 hours. We harvested cells by centrifuging them at 5,000 rpm for 15 minutes, and we froze cell pellets at -80°C for future use.

We purified proteins using a fast protein liquid chromatograph (FPLC, AKTA Pure). For most proteins, we lysed cell pellets by adding a standard lysis buffer: 4 mL of buffer (B-PER) with 2 mg TAME, 2 mg magnesium sulfate heptahydrate, 3.5 mg TCEP, 3.75 µl PMSF, 0.5 mg lysozyme, and 200 units DNase I (New England Biolabs)) per g of cell pellet. For proteins purified under denaturing conditions (all mutants of *Ec*FabG), we added 6M urea. And for mutants of *Pp*FabG4, which were poorly soluble, we used a special lysis buffer: 4mL of buffer (300mM NaCl, 50mM sodium phosphate, 20% glycerol, 1% Triton X-100, pH = 7.5) with 2 mg TAME, 2 mg magnesium sulfate heptahydrate, 3.5 mg TCEP, and 3.75 µl PMSF, 0.5 mg lysozyme, and 200 units DNase per gram of cell pellet.

We isolated histidine-tagged protein by flowing lysate over a nickel-affinity column (HisTrap HP column). For standard purifications, we used 50 mM Tris-HCl, pH 7.5, 0.5 mM TCEP, 300 mM NaCl, and 0-500 mM imidazole. For mutants of *Pp*FabG4, we used 50mM sodium phosphate, pH 7.5, 0.5 mM TCEP, 300mM NaCl, 20% glycerol, and 0-500mM imidazole. For mutants of *Ec*FabG, we used the "standard purification" procedure but added 6M urea to the running buffer and included an on-column protein refolding step. Briefly, before elution, we replaced the urea-including running buffer with urea-free buffer over a 1-hour gradient. For all proteins, we buffer-exchanged isolated protein with a spin concentrator (3-10 kDa VivaSpin; Sartorius) and purified it further, where necessary, with an anion-exchange column (HiPrep Q HP 16/10 column with 50 mM Tris-HCl, pH 7.5 or 8.5, 0.5 mM TCEP, and 0-1 M NaCl) or a cation-exchange column (HiPrep SP HP 16/10 column with 50mM sodium phosphate, 0.5 mM TCEP, 20% glycerol, and 0-1 M NaCl).

We used standard procedures to confirm and store purified proteins. We confirmed protein purity and molecular weight by using sodium dodecyl sulfate polyacrylamide gel

electrophoresis (SDS-PAGE), carried out a final buffer exchange, and concentrated purified proteins with a spin concentrator (5 or 10 kDa VivaSpin as before). For all proteins, we measured final concentrations with a Bradford assay by using bovine serum albumin (BSA) as a standard. We flash froze purified proteins and stored them in 20% glycerol at -80°C. For proteins of low abundance, we used only nickel-affinity chromatography, which was typically sufficient to achieve single-band purity with SDS-PAGE (e.g., *E. coli* FabZ).

In vitro Analysis of Fatty Acid Synthases. We characterized variants of FabH, FabG, and ACP by swapping them into the *in vitro* reconstituted FAS from *E. coli*. For all assays, we used the indicated concentration of protein under study (e.g., the x-axes in Fig. S13) in a standard reference background (100 µL; 1 µM all Fab enzymes, 10 µM 'TesA, 10 µM holo-ACP, 10 mM HEPES, pH 7.4, 150 mM NaCl, 0.5 mM TCEP) supplemented with 1.3 mM NADPH or NADH, 0.5 mM malonyl-CoA, and 100 µM acetyl-CoA or a specified alternative acyl-CoA. For mixtures containing octanoyl-CoA, we used 10 µM *Pp*FabH2 instead of 1 µM *Ec*FabH. After adding substrate, we monitored the conversion of NAD(P)H to $NAD(P)^+$ by measuring absorbance (340 nm) in a SpectraMax M2 plate reader, as described in prior work[1]. We converted the absorbance values to NADPH via a standard curve and estimated initial rates over 2.5 minutes. For each composition, we used at least three independently reconstituted reactions.

Extraction, Esterification, and Quantification of Fatty Acids. We quantified fatty acids in unknown samples of 400 µL reconstituted FAS reactions after 2 hours using the extraction, esterification and GC/MS analysis procedures described in prior work[2]. In brief, we extracted fatty acids with 200 µL ethyl acetate and 50 µL 10% NaCl (w/v) after quenching the reaction

with 50 µL of acetic acid and adding 20 µL 90.5 mg/L pentadecanoic acid as an internal standard. We vortexed the mixture for 10 s and centrifuged it for 10 min at 12,100g. We transferred 100 µL of the ethyl acetate layer to a clean glass vial and evaporated off the solvent. Next, we performed an hour-long methanol/HCl esterification by adding 400 µL of a 30:1 mixture of methanol and 37% (v/v) HCl in water, vortexing for 10 s and incubating at 50 °C for 1 hour. Finally, we added 200 µL of water and hexane, vortexed for 10 s, and transferred 100 µL of the hexane layer to a clean vial for GC/MS analysis, as described previously[2]. We calculated concentrations of each fatty acid using standard curves for each fatty acid species.

Kinetic Modeling Predictions. In prior work, we developed a kinetic model of the *E. coli* FAS that captures the activities of all nine enzymes used in our reconstituted in vitro system. This model uses mechanism-based rate equations and physiologically relevant concentrations of enzymes, substrates, and cofactors (i.e., 1 µM of each Fab enzyme, 10 µM TesA, 10 µM ACP, 100 µM acyl-CoA, 500 µM malonyl-CoA, 1 mM NADH, and 1 mM NADPH). In this study, we modified this model to include the activity of FabH variants on their preferred substrates. We parameterized EcFabH and PpFabH1 to act only on acetyl-CoA, and *Pp*FabH2 to act only octanoyl-CoA. For PpFabH1 and PpFabH2, which are new to this study, we fit the model to the experimental data in Figure 2B and 2C, respectively, by using the MATLAB function fminsearch to optimize the kcat for FabH. We used the MATLAB solver ode15s to solve the final kinetic model, applying relative and absolute tolerances of 10^{-6} and incorporating a vectorization step to reduce solve time. To calculate the error of the model, we randomly selected 300 concentrations of protein and substrate, separately, from normal distributions

centered at the designated concentrations with a standard deviation of 5% of the mean. We used the standard deviation of these runs as the model error (Fig, S4).

Crystallography. We prepared crystals of *Pp*FabG4 via hanging drop vapor diffusion. In brief, we added 1 µL of *Pp*FabG4 (~60 µM *Pp*FabG4, 50 mM Tris-HCl, pH 8.5) to 6 µL of crystallization solution (0.1 M Tris-HCl, 0.12 M ammonium chloride, pH 8.5, 20% PEG-4000) and incubated droplets over crystallization solution for 12 days at 20 °C (EasyXtal CrystalSupport, Qiagen). We prepared all crystals for freezing by soaking them in a 90/10 (v/v) mixture of buffer (0.1 M Tris-HCl, 0.12 M ammonium chloride, pH 8.5, 20% PEG-4000) and glycerol.

We collected X-ray diffraction data through the Collaborative Crystallography Program at Lawrence Berkeley National Lab (ALS ENABLE, beamline 8.2.2, 100 K, 1.00003 Å). In short, we performed integration, scaling, and merging of X-ray diffraction data using the xia2 software package, and we carried out molecular replacement (model, PDB entry 1Q7B) and structure refinement with the PHENIX graphical interface, supplemented with manual model adjustment in COOT[3] and one round of PDB-REDO[4]. for diagnostic purposes. Our crystal is reported in the RCSB Protein Data Bank (entry 9NG1) with statistics described in Table S6.

Sedimentation Velocity Analytical Ultracentrifugation (AUC). We examined the oligomeric state of *Pp*FabG4 with a standard experimental workflow. We prepared samples of *Pp*FabG4 WT at 102 µM and 38 µM in AUC buffer (40 mM NaPO4 pH 7.5, 100 mM NaCl, and 0.5 mM TCEP), loaded them into a standard two-channel Epon centerpiece containing AUC cells, and placed it into an An60Ti rotor. We collected data with a Beckman XL-A ultracentrifuge in

absorbance mode set to 280 nM and 50000 rpm at 20°C and carried out data processing with UltraScan3 v4.0 using a standard analysis pipeline[5]. In brief, we completed time and radially invariant noise and meniscus fitting via two- dimensional spectrum analysis (2DSA) followed by initial model generation using iterative 2DSA fitting. We used this model for genetic algorithm and Monte Carlo analysis (GA and MC-GA), used the final MC-GA model to determine sedimentation coefficients and molecular weight distributions, and carried out Van Holde-Weischet analysis in UltraScan3.

Molecular Dynamics Simulations. This study focuses on the impact of sequence differences in FabG on its substrate specificity, so we performed all experiments and simulations on *Ec*ACP, which is well characterized and extremely similar to *Pp*ACP1 (86% sequence identity). At the start of this study, no structures of the FabG-ACP complex were available, so we used a computational workflow to initialize the structures of *Ec*FabG and *Pp*FabG4 in complex with ACP. We started with apo structures of *Ec*FabG (PDB 1Q7B) and *Pp*FabG4 (PDB entry 9NG1) and extracted a structure of ACP from a crystal structure of *Ec*FabF bound to C16 acyl-ACP (PDB 6OKG). We chose to use the conformation of ACP in an enzyme-ACP complex rather than its conformation in an ACP crystal structure because the latter conformation has the acyl chain sequestered within ACP and is unique to the uncomplexed solution state of the enzyme. As a final note, structures of *Hp*FabG in complex with βkACPs were published during the course of our work, but we decided to use them as a final check on our workflow (Fig. S11), rather than as a guide that might introduce bias.

We searched for a stable binding site for holo-ACP by starting with *Ec*FabG. Using Rosetta[6], we identified 500 complexes with high docking scores and down selected to ten by

looking for conformations with three attributes consistent with catalytically competent binding: (i) ≤ 2.5 nm between S36 on ACP (i.e., the Ppant site) and both S138 and Y151 on FabG, (ii) at least one non-bonded interaction (< 4 Å) between ACP and the catalytic FabG, and (iii) a least three non-bonded interactions between ACP and other members of the FabG tetramer.

For the C10 substrate, a representative medium chain, we added the Ppant arm and acyl chain in segments. We used one segment for S36 and eight segments of 3-6 heavy atoms each for the acyl chain for a total of nine segments. We used OpenEye Omega[7] to generate conformers for each pair of main segments (i.e., for segments A-I, we generated conformers for AB, BC, CD, etc.) to yield 47, 4, 4, 35, 31, 200, 89, and 2 conformers. For each of the ten FabG / apo-ACP complexes generated by ROSETTA, we used an iterative algorithm to grow the acyl chain (see Fig. S17 for full description). At each step, this algorithm aligns a new segment to common atoms in the prior structure and eliminates any structures that clash with that structure (distance < 1 Å). We ensured growth into the binding pocket by eliminating any structures for which the terminal atom is farther from the catalytic residues than in previous iterations. Finally, we performed energy minimization using GROMACS on the complex to a threshold of 100 KJ/mol/nm and eliminated any structures for which the minimization is unable to converge. We performed this loop until all eight segments were added to the main chain. Only four of the ten original complexes allowed for growth of the entire acyl chain, yielding 47 FabG / β-ketoacyl-ACP (βkACP) complexes. The other 6 complexes reached a point where the chain could not be constructed without introducing steric clashes before chain completion. We then filtered with a 4 Å threshold distance to the catalytic residues S138 and Y150 to produce nine unique complexes.

We down selected the final nine structures with several additional steps. We subjected them to (i) an additional energy minimization with a 50 kJ/mol/nm threshold followed by

restrained NVT and NPT equilibration and (ii) a 50-ns MD simulation with a distance restraint applied between the β carbonyl and the donor hydrogens on residues S138 and Y151. This restraint applies no counter force when the interatomic distance is less than 3 Å, a harmonic restraint of $V = \frac{1}{2}k(r - 3\text{ Å})^2$ at distances between 3 and 5 Å, and a force given by $V = k\ (2r - 8\text{Å})$ for distances beyond 5 Å. This restrained simulation allows for the complex to stabilize without leaving a catalytically competent conformation. Finally, we carried out a 300-ns unrestrained MD simulation. Only one of the nine original conformations remained stable and in a catalytically competent conformation throughout the 300-ns unrestrained simulation. We used the centroid of the most populated cluster from this complex (i.e., the structure with the minimal heavy-atom RMSD to all other structures within the cluster) to generate conformations for C12, C14, and C16 complexes by using MODELLER 10.1 to grow the chain. When these conformations were used for short chain complexes (C4-C8), the short chains became unstable and forced NADPH out of the binding pocket, so we restarted our effort with C4 (Fig. S8).

For the C4 substrate, we generated 13 conformers using the procedure described above and subjected them to the same iterative growth protocol, energy minimization, restrained NVT and NPT equilibration, and catalytic distance restrains applied to C10. As before, only 1 conformation remained stable and in a catalytically competent conformation over the 300-ns MD simulation. We used the centroid of the most populated cluster from this complex to construct C6 and C8 initial configurations using MODELLER 10.1.

We estimated the stability of all final complexes by running unrestrained simulations in triplicate. For the second and third replicates, we randomly selected two initial structures from the equilibrated frames of the first replicate by using a random number generator and used 5-ns simulations with catalytic distance restraints to equilibrate the complex (which should already be

stable from our initial preparation). We then ran each unrestrained system for 500 ns of production simulation.

For *Pp*FabG4, we selected initial conformations by aligning the tetramer with the stable *Ec*FabG complexes for all chain lengths. After alignment, we confirmed that the distance between the β-carbonyl and reactive hydrogen on S138 and Y151 was less than 5 Å. We used the same protein structure for all three replicates, which only differ by the ACP conformation. The motivation for this choice was that the short chain complexes became too unstable, which did not allow us to use snapshots throughout the simulation without initiating a simulation with an unbound complex. To ensure that the production simulations were given the best chance of producing stable complexes we used 50 ns simulation with distance restraints for each of the three replicates at each of the three chain lengths (4C, 6C, 8C, 10C, 12C, 14C, and 16C), just as was done with replica 1 for the *Ec*FabG complex simulations. These restrained simulations also introduce increased diversity in replica conformations when initiating production simulations.

To examine the effect of mutations on *Ec*FabG or *Pp*FabG, we used MODELLER 10.1 to introduce mutations into wild-type configurations, and we equilibrated the resulting complexes for 50 ns with catalytic distance restraints as above. For all complexes which were stable in WT simulations (this excludes *Pp*FabG4 with short chain substrates), the mutation was applied to the centroid structure from the WT simulations for each of the three replicas. For *Pp*FabG4 with short chain substrates we applied the mutations to the conformations produced at the end of the 50 ns restrained simulation of the WT complexes since stable complexes were not produced by the unrestrained simulations. As with the WT simulations, following 50 ns restrained equilibration we ran all simulations for 500 ns per simulation. The initial configurations and topologies for all production simulations can be found at github.com/shirtsgroup/FAS.

We used the same parameterization protocol for all molecules. We determined protein protonation states by using the H++ web server, parameterized all protein residues and NADPH with the Amber ff14sb force field, and parameterized the full β-ketoacyl chain (Ppant and β ketoacyl segments combined) with the GAFF 2 force field[8]. To speed up our simulations, we applied hydrogen mass repartitioning to all topologies by using ParmED with hydrogen mass set to 3AU, which allows for a 3 fs timestep. We constructed a periodic box as a dodecahedron with a minimum distance to the wall of 1 nm from the protein and filled this box with TIP3P solvent and a salt concentration of 0.15M[9]. We performed all simulations at 300 K using the Bussi-Parrinello thermostat and 1 atm using the stochastic cell rescaling barostat[10,11]. We ran all simulations using GROMACS 2021.5[12].

Following completion of all production simulations, we applied a standard trajectory processing procedure using the GROMACS *trjconv* function, which clusters the full protein complex to keep it together across periodic boundaries and removes the rotational and translational motions of the complex. We defined the equilibration point for each trajectory as the frame in which the heavy atom RMSD for the reactive FabG monomer and ACP are stable within a 2 Å threshold. We used only the trajectory frames after the equilibration point for clustering and further analysis. We applied RMSD clustering by using the GROMACS *cluster* function on the backbone atoms of the catalytic FabG and ACP proteins and used the centroid of the most populated cluster as the reference for any RMSD or RMSF calculations.

In our analyses, we define a catalytically competent FabG-ACP complex (as opposed to a catalytically competent FabG conformation) as one that satisfies three criteria: (i) at least two interactions form between FabG and ACP (i.e., the heavy atom distance between a residue on ACP and a residue on any FabG monomer is less than 4 Å), (ii) the distances between each

donor hydrogen on S138 and Y151 and the β carbonyl is less than 3Å, and (iii) the distance between the donor hydrogen on NADPH and the β carbonyl is less than 5Å. The percent catalytic competency reported in this paper is the percent of equilibrated frames in a given trajectory for which the complex satisfies these three criteria.

We also analyzed non-bonded interactions between FabG monomers and the ACP, Ppant, and β ketoacyl chain in this study. Non-bonded interactions are heavy atom distances of less than 4 Å. For inter-residue interactions, we consider the minimum heavy atom distance for all heavy atoms in each residue; for interactions within the Ppant and β ketoacyl chain we also examined interaction with individual heavy atoms rather than treating the Ppant and β ketoacyl chain as a single residue. We also computed the surface area of the ACP-FabG interface from the perspective of each member of the FabG tetramer. We used the MDTraj function[13] to estimate the solvent accessible surface area (SASA) for (i) each unit of the tetramer when bound to one ACP and (ii) each unit of the tetramer with no ACP present.

SI NOTES

Note 1. Bioinformatic analysis of FAS-like genes in *Pseudomonas putida* (*P. putida*) *KT2440*.

KT2440 contains a type II fatty acid synthase and other FAS-like enzymes with unknown functions (Table S1). The FAS has a malonyl-CoA:ACP transacylase (FabD; PP_1913), β-ketoacyl-ACP synthase III (FabH; PP_4379), β-ketoacyl-ACP reductase (FabG; PP_1914), β-hydroxyacyl-ACP dehydratase (FabZ, PP_1602), β-hydroxydecanoyl-ACP dehydratase (FabA; PP_4174), enoyl-ACP reductase (FabV; PP_4635), β-ketoacyl-ACP synthase II (FabF; PP_1916), β-ketoacyl-ACP synthase I (FabB; PP_4175) and an acyl carrier protein (ACP; PP_1915). In total, we found nine KSs, six oxidoreductases—four β-ketoacyl-ACP reductases (KRs) and two putative enoyl-acyl-ACP reductases (ERs)— and two ACPs (Fig. 1B, Table S1). The auxiliary ACP, one auxiliary β-ketoacyl-ACP reductase, and four auxiliary β-ketoacyl-ACP synthases are located next to each other within the *P. putida KT2440* genome (PP_2777-PP_2783), indicating the presence of a potential gene cluster, and thus, related function.

Note 2. Analysis of initiation activity. The high FAS activity conferred by EcFabH and FabH1, a putative analogue, on octanoyl-CoA in main text Fig. 2B is inconsistent with structural studies showing sub-optimal binding of long-chain precursors to EcFabH (e.g., a specific activity on hexanoyl-CoA over ten-fold smaller than that observed on acetyl-CoA[15,16]). We speculated that short chain acyl-CoA substrates (e.g., acetyl-CoA) for EcFabH and FabH1 might emerge from KS-catalyzed decarboxylation of malonyl-CoA or malonyl-ACP. To explore this hypothesis, we carried out assays in the absence of acyl-CoA substrates (Fig. 2C). Indeed, the activity of EcFabH and FabH1 was preserved (Fig. 2C), an indication of acyl-CoA substrate production by another enzyme (i.e., FabF and/or FabB). Subsequent modeling suggests that FabF and FabB can facilitate an alternative initiation route where either enzyme can decarboxylate malonyl-ACP into acetyl-ACP followed by FabB-catalyzed transacylation to produce acetyl-CoA from acetyl-ACP (Table S5, Fig. S4). Previous studies of E. coli KSs have suggested that FabF and FabB can also catalyze the direct condensation of malonyl-ACP and acetyl-ACP in the absence of FabH[2,14]. Our experimental results indicate that this activity is negligible; nonetheless, modeling results show similar trends with and without it, suggesting that acetyl-CoA production by FabB and FabF is the primary cause of the activity of EcFabH and FabH1 observed in the absence of acetyl-CoA.

Note 3. Simulated error of the Bradford assay. The Bradford assay is an inherently inaccurate approach for determining enzyme concentration. To investigate the impacts of this imprecision on our results, we used the kinetic model to simulate the effects of high error in enzyme concentration (Fig. S4). In brief, we simulated the impact of 30% error in enzyme concentration by sampling from a normal distribution of concentrations, and we evaluated the impact of this error on our results. Although this level of variability results in a substantially larger standard error in the initial rate, the overall trends remain consistent, as evidenced by comparison between A-C and D-F in Fig. S4. This analysis, alongside the additivity of mutational effects, suggests observed differences in the substrate specificities of enzyme variants cannot be explained by errors in enzyme concentration.

Note 4. Results of sedimentation velocity analytical ultracentrifugation. We performed sedimentation velocity analytical ultracentrifugation to examine the oligomeric state of *Pp*FabG4 in solution at both a high concentration, 102 µM, and a low concentration, 38 µM. At both concentrations *Pp*FabG4 existed in one dominate oligomeric state, evidenced by the narrow distribution of sedimentation coefficients in van Holde-Weischet analysis. While the low concentration *Pp*FabG4 sample does have a wider range this is likely a result of low signal as is the lack of robust statistics from Monte Carlo analysis. Experimentally determined molecular weights, 98.6 kDa and 101.4 kDa for the high and low concentration respectively, support that *Pp*FabG4 exists as a tetramer (predicted molecular mass 113.9 kDa) in solution.

SI FIGURES

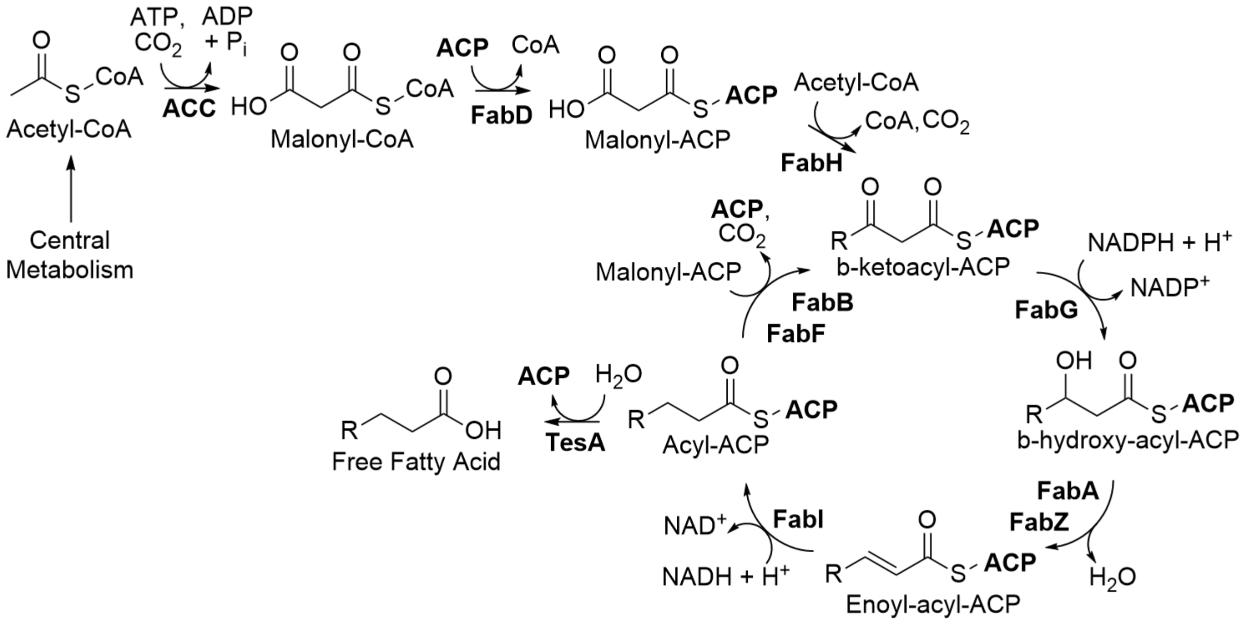

Figure S1. The fatty acid synthase of E. *coli*. The complete fatty acid synthase (FAS) from *E. coli*. Enzymes: acetyl-CoA carboxylase (ACC), fatty acid:CoA ligase (FabD), β-ketoacyl-ACP synthase III (FabH), β-ketoacyl-ACP reductase (FabG), β-hydroxyacyl-ACP dehydratase (FabZ), β-hydroxy-decanoyl-ACP dehydratase (FabA), enoyl-ACP reductase (FabI), β-ketoacyl-ACP synthase I (FabB), β-ketoacyl-ACP synthase II (FabF), and thioesterase 1 (TesA).

Identified secondary metabolite regions using strictness 'loose'

NC_002947.4 (Pseudomonas putida KT2440)

Region	Type	From	To	Most similar known cluster		Similarity
Region 1	saccharide	406,364	425,309	lipopolysaccharide	Saccharide	27%
Region 2	redox-cofactor	446,620	468,779	lankacidin C	NRP+Polyketide	13%
Region 3	saccharide	580,711	600,653			
Region 4	saccharide	969,088	1,003,979	gamexpeptide A/gamexpeptide B/gamexpeptide E/luminmide B/luminmide D/luminmide E/luminmide F/luminmide G	NRP	18%
Region 5	saccharide	1,509,749	1,531,701			
Region 6	NAGGN	1,941,555	1,956,445			
Region 7	saccharide	1,973,991	2,049,271	lipopolysaccharide	Saccharide:Lipopolysaccharide	25%
Region 8	fatty_acid	2,151,317	2,169,636			
Region 9	saccharide	2,852,294	2,885,878			
Region 10	RRE-containing	3,060,840	3,080,793			
Region 11	fatty_acid	3,153,797	3,177,762	koreenceine A/koreenceine B/koreenceine C/koreenceine D	Polyketide	100%
Region 12	saccharide	3,528,205	3,567,447	exopolysaccharide	Saccharide	12%
Region 13	saccharide	3,680,758	3,702,567	MA026	NRP	2%
Region 14	fatty_acid	3,729,073	3,749,458	oxalomycin B	NRP+Polyketide	6%
Region 15	ranthipeptide, halogenated	3,913,383	3,941,107	Pf-5 pyoverdine	NRP	8%
Region 16	RiPP-like	4,111,172	4,120,928			
Region 17	saccharide	4,554,991	4,595,084	pseudopyronine A/pseudopyronine B	Other:Fatty acid	62%
Region 18	fatty_acid	4,708,259	4,727,381			
Region 19	NRP-metallophore, NRPS, RiPP-like	4,738,161	4,851,189	Pf-5 pyoverdine	NRP	18%
Region 20	fatty_acid	4,961,082	4,980,561			
Region 21	fatty_acid	5,152,678	5,173,799			
Region 22	saccharide	5,598,326	5,635,760			
Region 23	RiPP-like	5,969,781	5,980,056			

Figure S2. Gene clusters from *Pseudomonas putida KT2440*. We used AntiSmash 7.0 to identify biosynthetic gene clusters in the genome of *P. putida* KT2440. The cluster encoded by genes PP_2777-2787 matches a cluster that encodes polyketide synthase from *Pseudomonas koreensis* that produces koreenceine analogs A-D (Fig. S3[15].

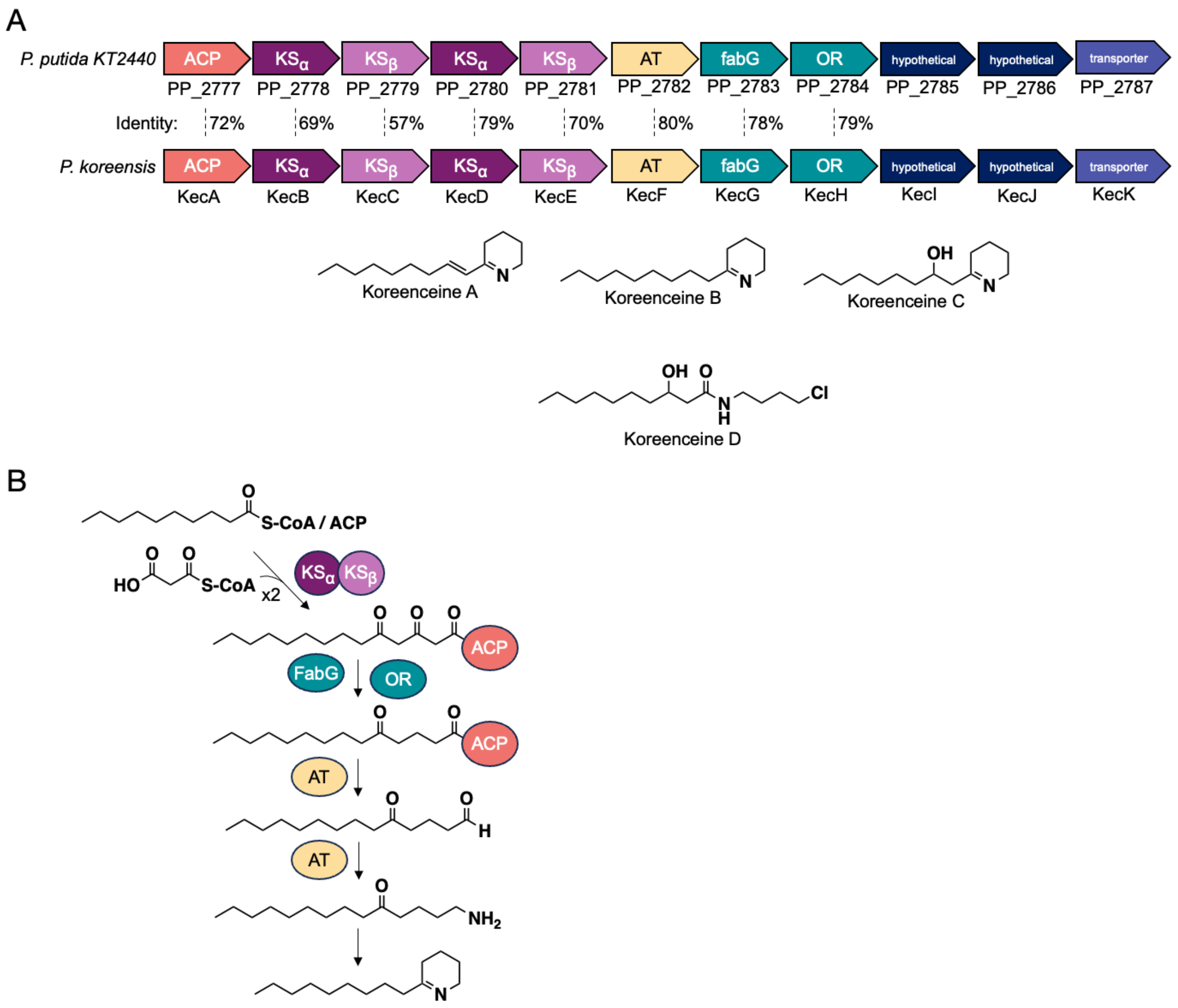

Figure S3. Gene cluster PP_2777-2784 encodes a type II PKS. (A) A comparison of biosynthetic gene clusters from *P. putida KT2440* and *P. koreensis*, the latter of which encodes a type II polyketide synthase that makes four koreenceine analogues[16]. This PKS is predicted to include genes PP_2777-PP_2784 in *P. putida* and BOW65_RS02920-RS02955 in *P. koreensis.* (B) The proposed pathway for koreenceine B synthesis is based on the predicted pathway for γ-coniceine synthesis, a koreenceine analog produced in plants[16].

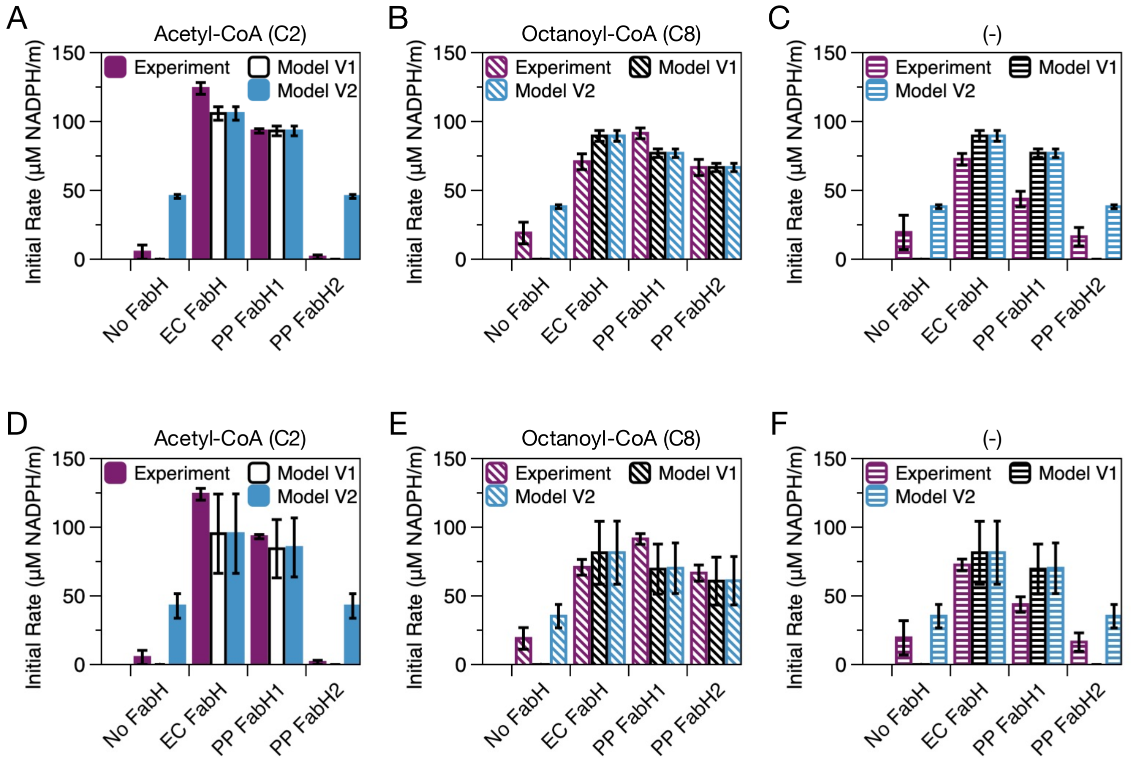

Figure S4. **Kinetic modeling of FabB-catalyzed decarboxylation of malonyl-CoA**. Initial rates of fatty acid synthesis by reconstituted *E. coli* FASs with variants of FabH and either (A) acetyl-CoA, (B) octanoyl-CoA, and (C) no acyl-CoA. Purple bars show experimental results (Figs. 2A-2C); black and white and blue bars show modeling results. We used a base model that includes FabF- and FabB-catalyzed decarboxylation of malonyl-ACP and condensation of malonyl-ACP with acetyl-ACP as described previously (SI Note 2, Table S5)[2]. Model V1, which is more consistent with the activity of *Pp*FabH2 on acetyl-CoA, removes the condensation activity of FabF and FabB; Model V2, which is more consistent with the observed activity of *Pp*FabH2 with no acyl-CoA substrate (other than malonyl-CoA) includes these mechanisms. All *in vitro* compositions include 1 µM of each FabH variant, 10 µM 'TesA, 10 µM holo-ACP, 1 µM of all other FAS enzymes, 1.3 mM NADPH, 0.5 mM malonyl-CoA, and 100 µM acyl-CoA, when included. Modeling results contain 1 µM of each FabH variant, 10 µM 'TesA, 10 µM holo-

ACP, 1 μM of all other FAS enzymes, 1 mM NADPH, 1 mM NADH, 0.5 mM malonyl- CoA, and 100 μM acyl-CoA, when included. Experimental data were collected over 2.5 minutes (initial rate) represent the mean and SE of $n \geq 3$ technical replicates. Modeling results were calculated after 2.5 minutes. The error bars depict SD for 300 runs using randomly chosen enzyme concentrations sampled from their normal distributions with standard deviations of (A-C) 5% and (D-F) 30% of their respective means; for substrate, we used 5% for all simulations. We used a high enzyme SD to simulate potential error inherent to the Bradford assay (SI Note 3).

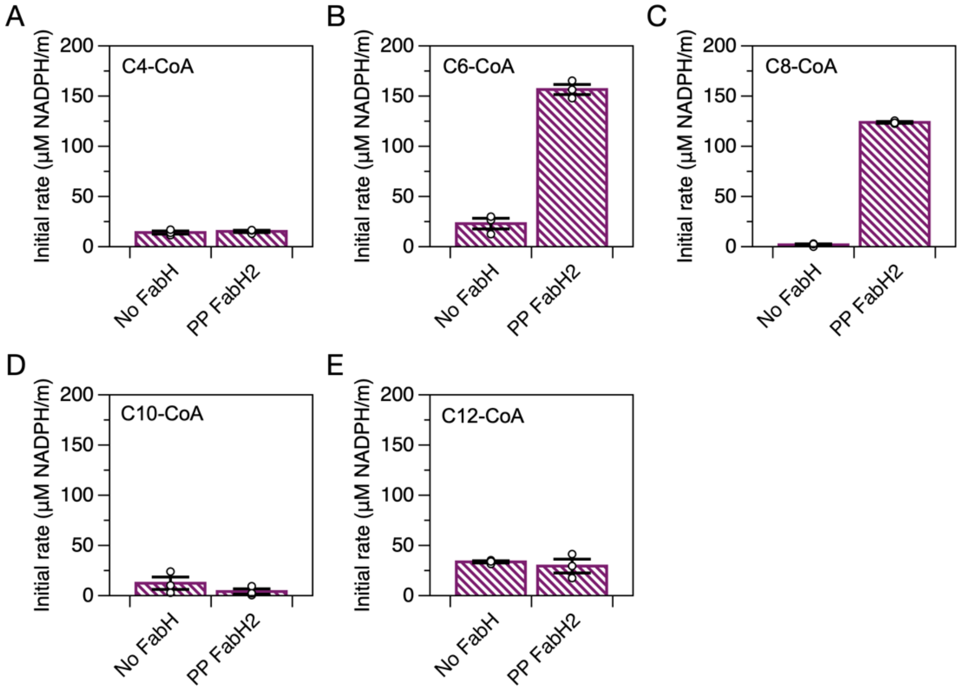

Figure S5. Chain length specificity of *Pp*FabH2 on acyl-CoAs. (A-D) The initial rate of fatty acid synthesis (2.5 min) of experimentally reconstituted FASs containing no FabH or 10 µM PP_4545 (PP_FabH2), 1 µM of all other *E. coli* Fab enzymes, 10 µM 'TesA (a leaderless variant of thioesterase I of *E. coli*), and 10 µM EC_ACP (holo-ACP from *E. coli*). All systems included 1.3 mM NADPH, 0.5 mM malonyl-CoA, and either 0.1 mM (A) butyryl-CoA (C4-CoA), (B) hexanoyl-CoA (C6-CoA), (C) octanoyl-CoA (C8-CoA), (D) decanoyl-CoA (C10-CoA), or (E) dodecanoyl-CoA (C12-CoA). Data represent the mean and SE of n = 3 technical replicates.

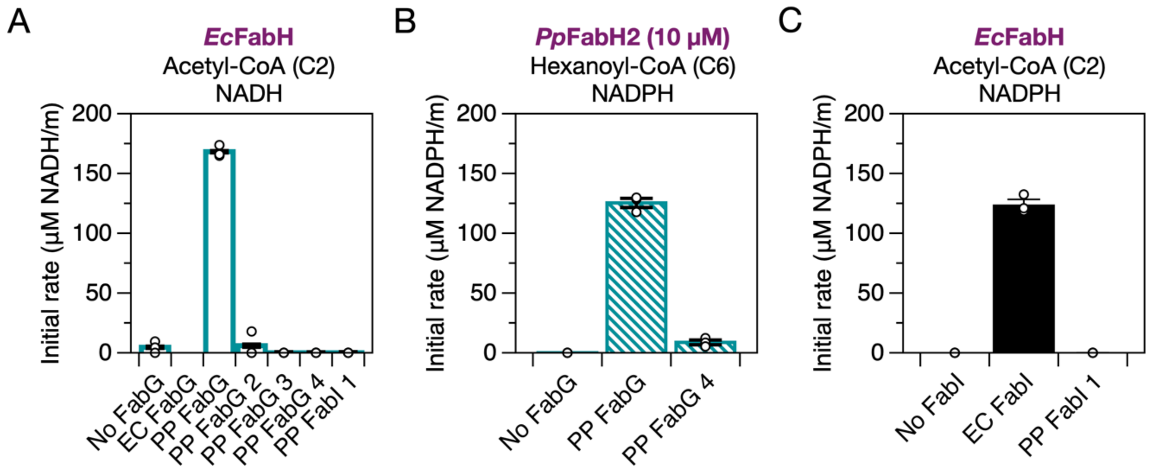

Figure S6. Analysis of β-ketoacyl-ACP reductases from *Pseudomonas putida KT2440*. The initial rate of fatty acid synthesis (2.5 min) of experimentally reconstituted FASs. All systems include 1 µM of each FabG variant or FabI variant, 1 µM of each other FAS enzyme (i.e., *Ec*FabD, *Ec*FabH, *Ec*FabA, *Ec*FabZ, *Ec*FabB, and *Ec*FabF), 10 µM 'TesA, 10 µM holo-ACP, 0.5 mM malonyl-CoA, 100 µM acyl-CoA, and specified cofactor concentrations. (A) Initial rates conferred by each of five oxidoreductases from KT2440 in place of *Ec*FabG in the presence of 1.3 mM NADH (instead of NADPH, Fig. 2) and acetyl-CoA (C2). (B) Initial rates conferred by two select KRs in place of *Ec*FabG in the presence of 1.3 mM NADPH and hexanol-CoA (C6). (C) Initial rates conferred by PP_1852 in place of *Ec*FabI in the presence of 1.3 mM NADPH and acetyl-CoA (C2). Data in A-C represents the mean and SE of n = 3 biological replicates.

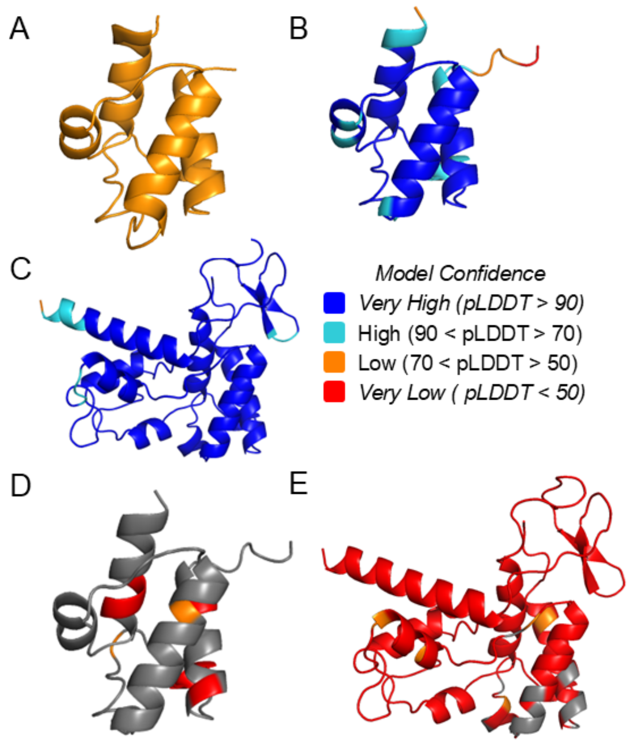

Figure S7. Structural analysis of ACPs from *P. putida* KT2440. (A) Crystal structure of ACP from *E. coli* (PDB 6OKG). (B-C) Structures predicted by AlphaFold3 for (B) PP_1915 and (C) PP_2777 with residues colored based on pLDDT, a per-residue measure of local confidence[17,18]. (D—E) We color (D) PP_1915 and (E) PP_2777 based on their sequence similarity to *E. coli* ACP. Residues are colored based as follows: (gray) identical, (orange) similar (e.g., valine and leucine), and (red) significantly different.

A

PPFabG4 WT	Monomer	Dimer	Tetramer
Predicted Molecular Mass (Da)	28,479	56,958	113,916

B C

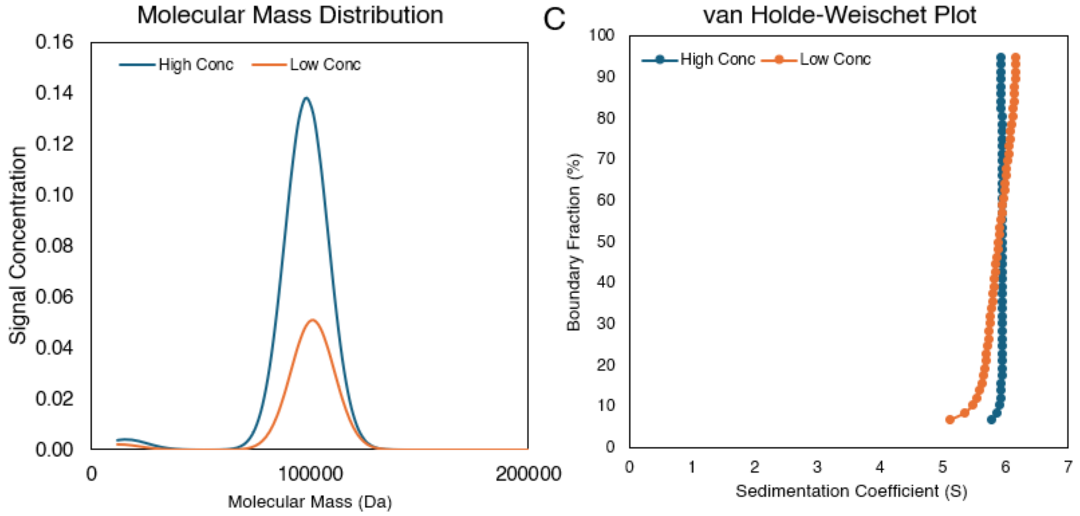

D

Sample Concentration	Sedimentation Coff (S)	f/f0	Determined Molecular Mass (Da)
High Conc: 102uM	5.6 (95% CI 5.5, 5.6)	1.25 (95% CI 1.24, 1.26)	98648 (95% CI 97539, 99756)
Low Conc: 38uM	6.0	1.28	101411

Figure S8. Sedimentation velocity analytical ultracentrifugation of *Pp*FabG4. (A) Table depicting the molecular weight of the monomeric *Pp*FabG4 purified construct along with predicted dimeric and tetrameric masses. (B) Molecular mass distribution for samples of *Pp*FabG4 at 102 µM (High Conc) and 38 µM (Low Conc). (C) van Holde-Weischet Plot depicting sedimentation coefficient distributions for both samples. (D) Table summarizing results showing determined sedimentation coefficients, frictional ratios, and molecular masses. Confidence intervals were through MC-GA. See methods for more information.

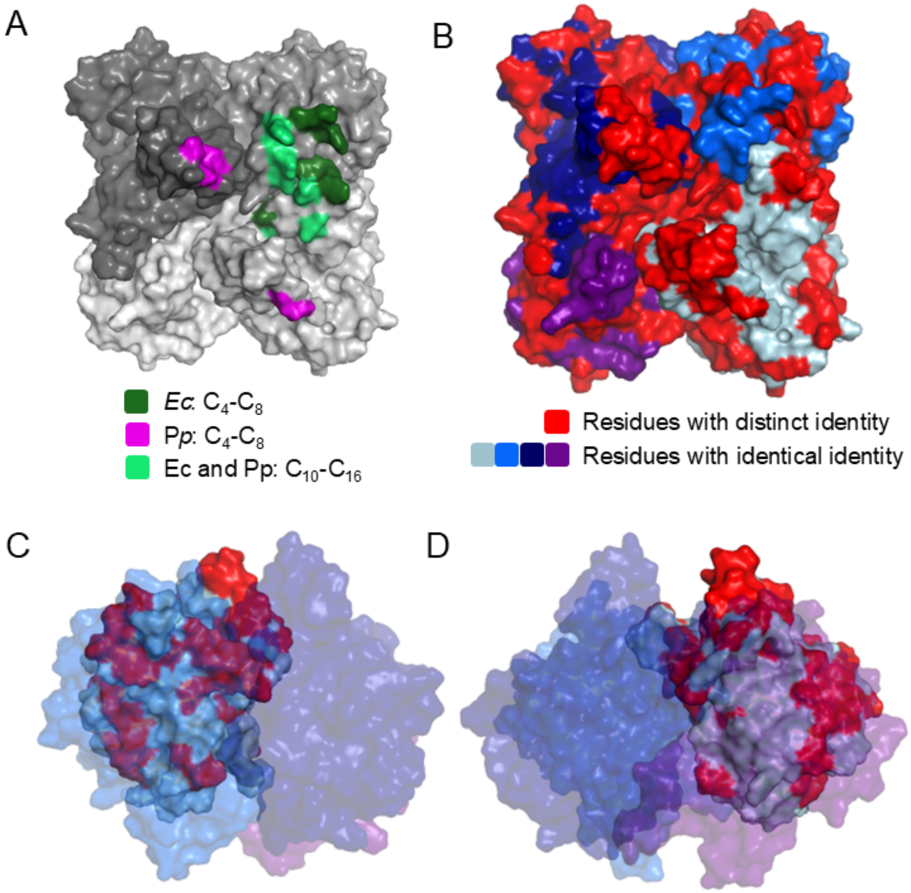

Figure S9: Sequence comparison between *Ec*FabG and PP_*Pp*FabG4. (A) Interaction interface for *Ec*FabG and *Pp*FabG4 for short and long chain substrates are found along the trimer interface. (B) A surface representation of *Pp*FabG4 shows regions that make non-bonding interactions with ACP for (dark green) *Ec*FabG bound to C4-C8, (pink) *Pp*FabG4 bound to C4-C8, and (light green) both EcFabG and *Pp*FabG4 bound to C10-C16. (B-D) Red highlights show residues with different identities between *Ec*FabG and *Pp*FabG4; all others appear in blue or purple. (C) Sequence differences localize near the binding interface for short chain substrates in *Ec*FabG and in outward facing residues. There are very few residue differences located within the acyl binding pocket (Fig. 3). (D-E) We also observe significant sequence differences at the interface between monomers which may also explain the lack of stability of the PP_2783 trimer complex.

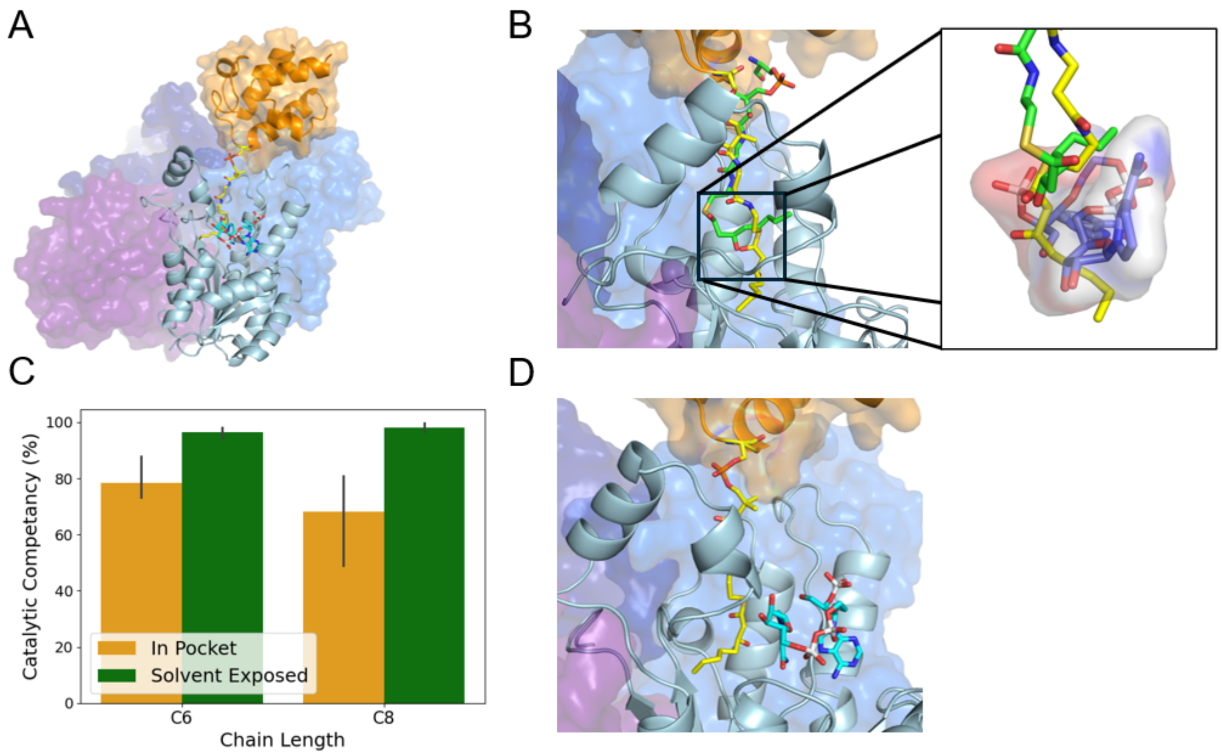

Figure S10. MD simulations of *Ec*FabG bound to short-chain acyl-ACPs. (A) A centroid showing the *Ec*FabG tetramer bound to C8-ACP; we initiated these simulations with the acyl chain in the acyl binding pocket. We define the "in- pocket" conformations as those that enable non-bonded interactions between the acyl chain and non-solvent exposed residues of the acyl binding pocket, primarily those in β1 (Figure S13), and the "solvent- exposed" conformations as those with a solvent exposed acyl chain. The C6 and C8 substrates had distinct in-pocket and solvent-exposed conformations; the C4 chain was too short for a distinct in-pocket conformation. (B) A comparison of a C8 acyl chain for both conformations. In the in-pocket conformation (yellow), the acyl chain wraps underneath NADPH, destabilizing it; in the solvent-exposed conformation (green), it curves around the binding location for NADPH. (C) The catalytic competency (defined by the presence of non-bonded interactions between FabG and ACP as well as distances of less than 3Å between the acyl chain carbonyl and the donor atom of S138 and Y151 and less than 5 Å from the donor atom in NADPH) of 100-ns simulations of the in-pocket

conformation compared to 500-ns simulations of the solvent-exposed conformation. We see a significant reduction in the catalytic competency for C6 and C8 complexes when the acyl chain is initiated in the binding pocket, rather than out of it. We plot the mean for n=3 simulations and error bars represent the standard error of the mean. (D) A representative snapshot from simulations initiated with acyl chain in an in-pocket conformation. In all three replicates, NADPH destabilized, increased pocket solvent exposure, and destabilized the acyl chain.

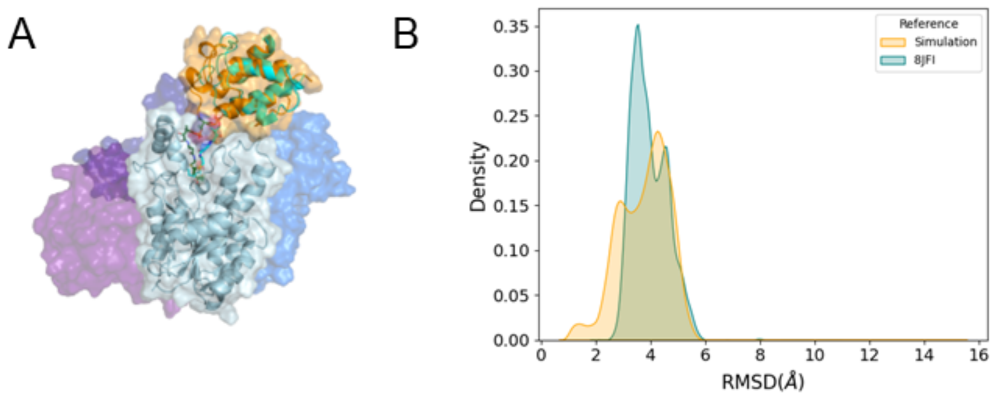

Figure S11: Comparison of the crystal structure of *Hp*FabG (PDB 8JFI) and a simulated complex of *Ec*FabG. (A) Structures of β-ketoacyl-hexanoyl ACP (C6βkACP) from (orange) MD simulations carried out with the *Ec*FabG tetramer and (turquoise) the X-ray crystal structure of the *Hp*FabG tetramer bound to C6βkACP. We compared these complexes by aligning the catalytic FabG monomer. The *Ec*FabG tetramer appears in blue and purple. (B) We compared the RMSD of ACP backbone atoms computed for our trajectories of *Ec*FabG bound to C6βkACP to both (orange) the centroid of those trajectories and (teal) the crystal structure of *Hp*FabG bound to C6βkACP. The plotted distributions correspond to n=3 simulations of the C6βkACP-*Ec*FabG complex. We do not see a significant difference (p=0.108 with 9619 uncorrelated samples) between the two distributions, an indication that the ACP conformation and binding site determined from our computational workflow is similar to the *Hp*FabG-ACP crystal structure.

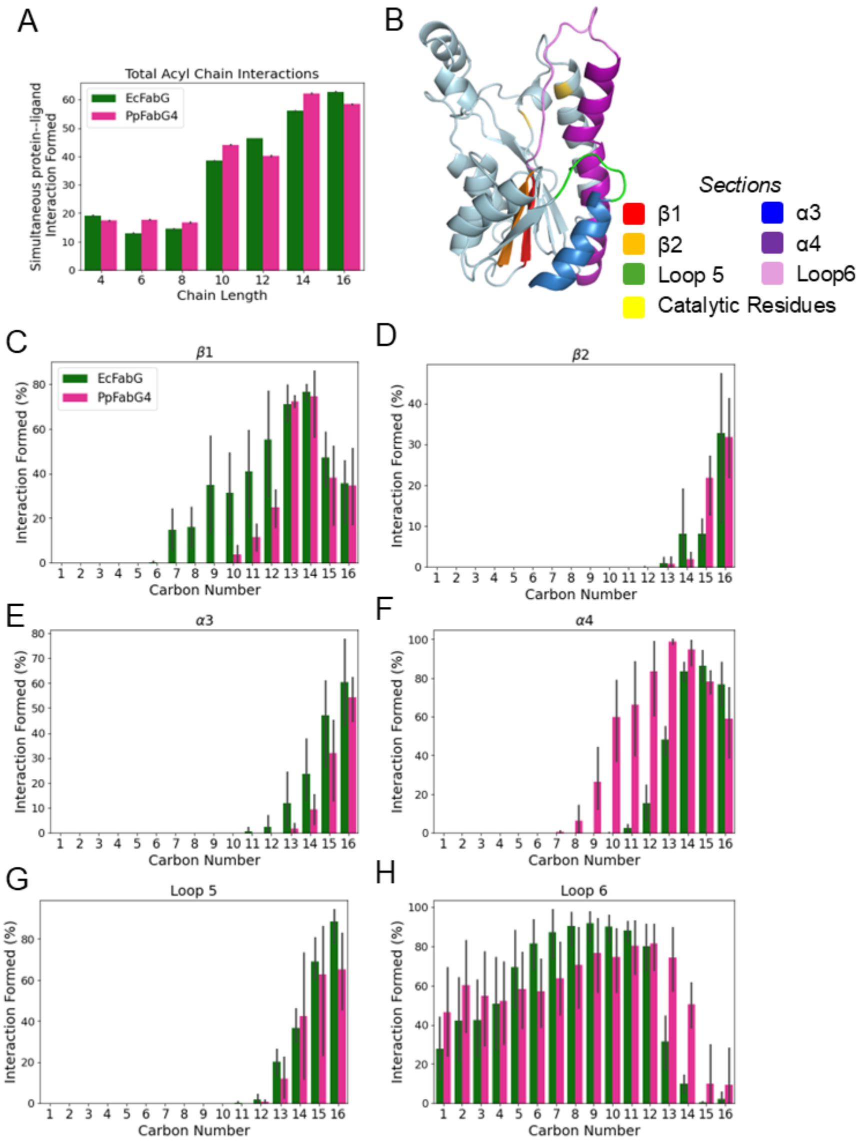

Figure S12: Comparison of acyl chain interactions within the binding pocket for *Pp*FabG4 and EcFabG. (A) The total number of simultaneous non-bonded interactions formed with the acyl chain shows clear divisions between chain lengths. For short chains (length 4-8) we observe a consistent number of weak non-bonded interactions, but for medium and long chains (length 10-16) we observe a two-fold jump in the number of interactions, which continues to increase with chain length**.** This plot shows the mean of n=3 simulations for each substrate. (B) To

investigate the locations of key interactions, we separated the acyl binding pocket into distinct regions. For each, we averaged the frequency of non-bonded interactions with each carbon (CN). (C) The β1 region forms interactions with carbons proximal to CN 10, but these interactions are not sufficient to stabilize acyl chains in the binding pocket of *Pp*FabG4, based on simulations of shorter chains (Fig. 4). (D-G) The β2, α2, and α4 regions form increasingly stable interactions farther into the chain (C10 to C16). (H) Only loop 6 can form any stable non-bonded interactions with carbons 1-8. (C-H) For CN 1-10, we used the mean from n=3 simulations for C10, C12, C14, and C16 substrates (n=12); for CN11 and CN12, we used the mean from simulations of C12, C14, and C16 (n=9); for CN12 and CN14, we used the mean of C14 and C16 simulations (n=6); and for C14 and C16, we used the mean of C16 substrate simulations (total of n=3). The error bars in each plot represent the standard error on the mean.

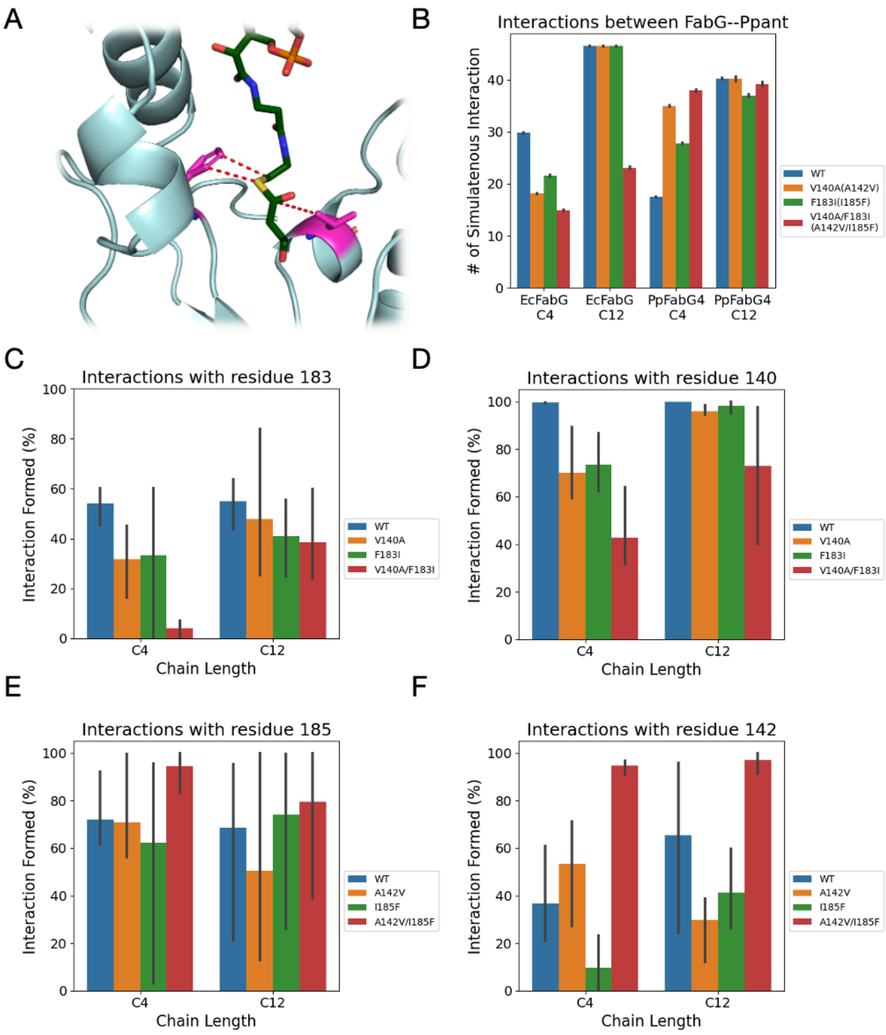

Figure S13. Contribution of the Ppant arm to the stability of the FabG-ACP complex. (A) Structure of *Ec*FabG bound to C4 βkACP, depicted as the centroid of MD simulations. Dashed lines depict non-bonded interactions between F183 and V140 on *Ec*FabG and the Ppant linker of βkACP. (B) We show the total number of interactions between the Ppant linker and the catalytic FabG monomer. Mutations appear for *Ec*FabG (*Pp*FabG4). In *Ec*FabG, the F183I and V140A mutations destabilize this interface; in *Pp*FabG4, the complementary substitutions (I185F and A142V) stabilize it. We also plot the direct non-bonded interactions formed with residues (C) 183 (C) and (D) 140 for *Ec*FabG and (E) 185 and (F) 142 in *Pp*FabG4. These residues strengthen the FabG-Ppant interface through the formation of non-bonded interactions with the Ppant linker.

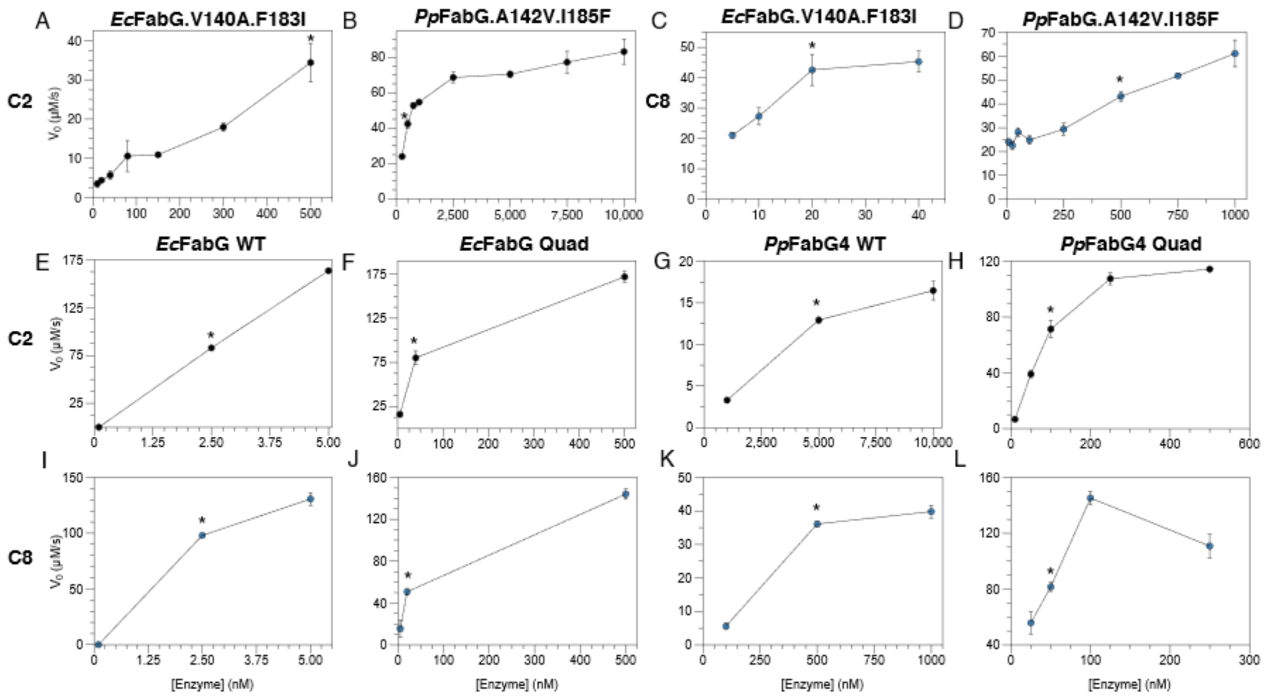

Figure S14. Determination of the linear range for FabG variants. We determine the enzyme concentration at which variants of FabG are rate limiting by reconstituted *E. coli* FASs with variants of (A, C) *Ec*FabG.V140A.F183I, (B, D) *Pp*FabG.A142V.I185F, (E, I) *Ec*FabG WT, (F, J) *Ec*FabG Quad, (G, K) *Pp*FabG4 WT, and (H, L) *Pp*FabG4 Quad with (A-B, E-H) acetyl-CoA or (C-D, I-L) octanoyl-CoA. Starred points represent selected rate-limiting concentrations (listed in Table S4) within the linear response range that were used for reconstituted *E.* coli FAS in Figures 3G-3H and 5E. FAS compositions: various concentrations of FabG, 1 µM *Ec*FabH or 10 µM *Pp*FabH2, 10 µM 'TesA, 10 µM holo-ACP, 1 µM of all other FAS enzymes, 1.3 mM NADPH, 0.5 mM malonyl-CoA, and 0.1 mM acyl-CoA. Measurement time: 2.5 minutes. Data depicts the mean and SE of $n \geq 2$ technical replicates.

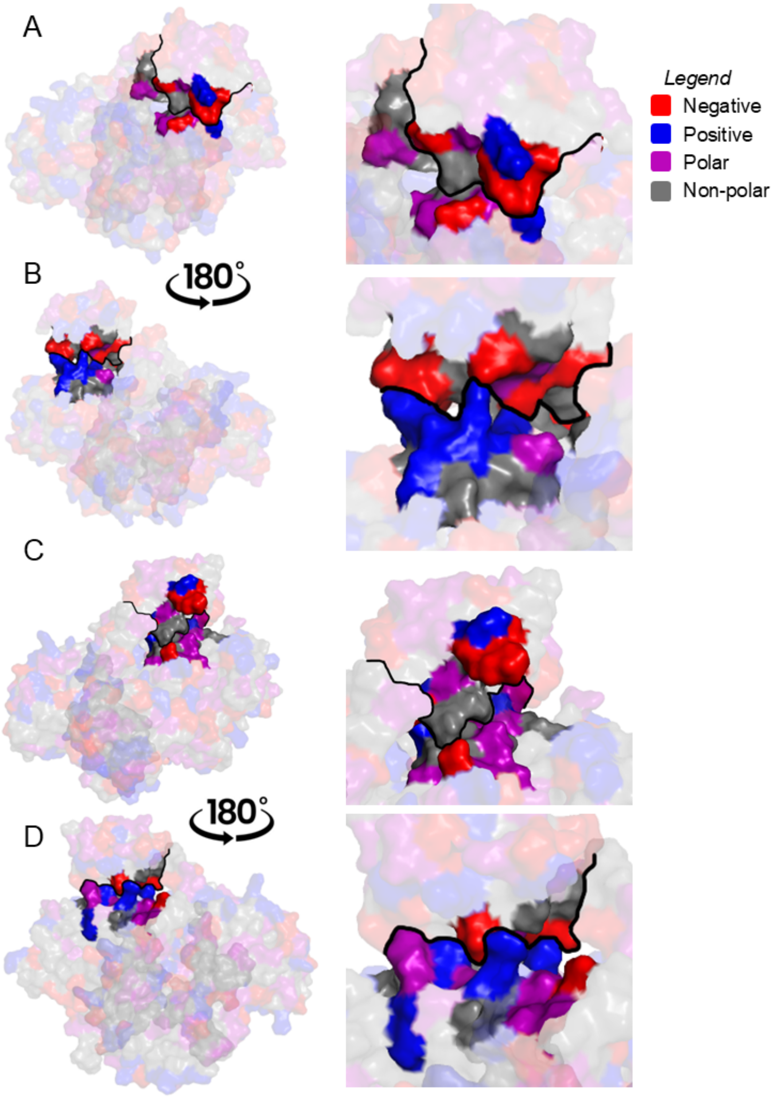

Figure S15: MD simulations of the FabG-ACP interfaces for *Ec*FabG and *Pp*FabG4. Structures of the FabG-ACP binding interface depicted as the centroids of MD simulations of (A-B) EcFabG bound to C4 βkACP and (C-D) PpFabG4 bound to C16 βkACP. Colors denote chemical functionalities of amino acids: negatively charged (red), positively charged (blue), polar (purple), or non-polar (gray). Semi-transparent residues do not directly participate in the FabG-ACP interface. Both *Ec*FabG and *Pp*FabG4 have patches of positive residues that align with patches of negatively charged residues on ACP. The positively charged patch in *Ec*FabG

contains more residues and a larger surface area resides on ACP, but this positively charged patch is larger in *Ec*FabG than in *Pp*FabG4.

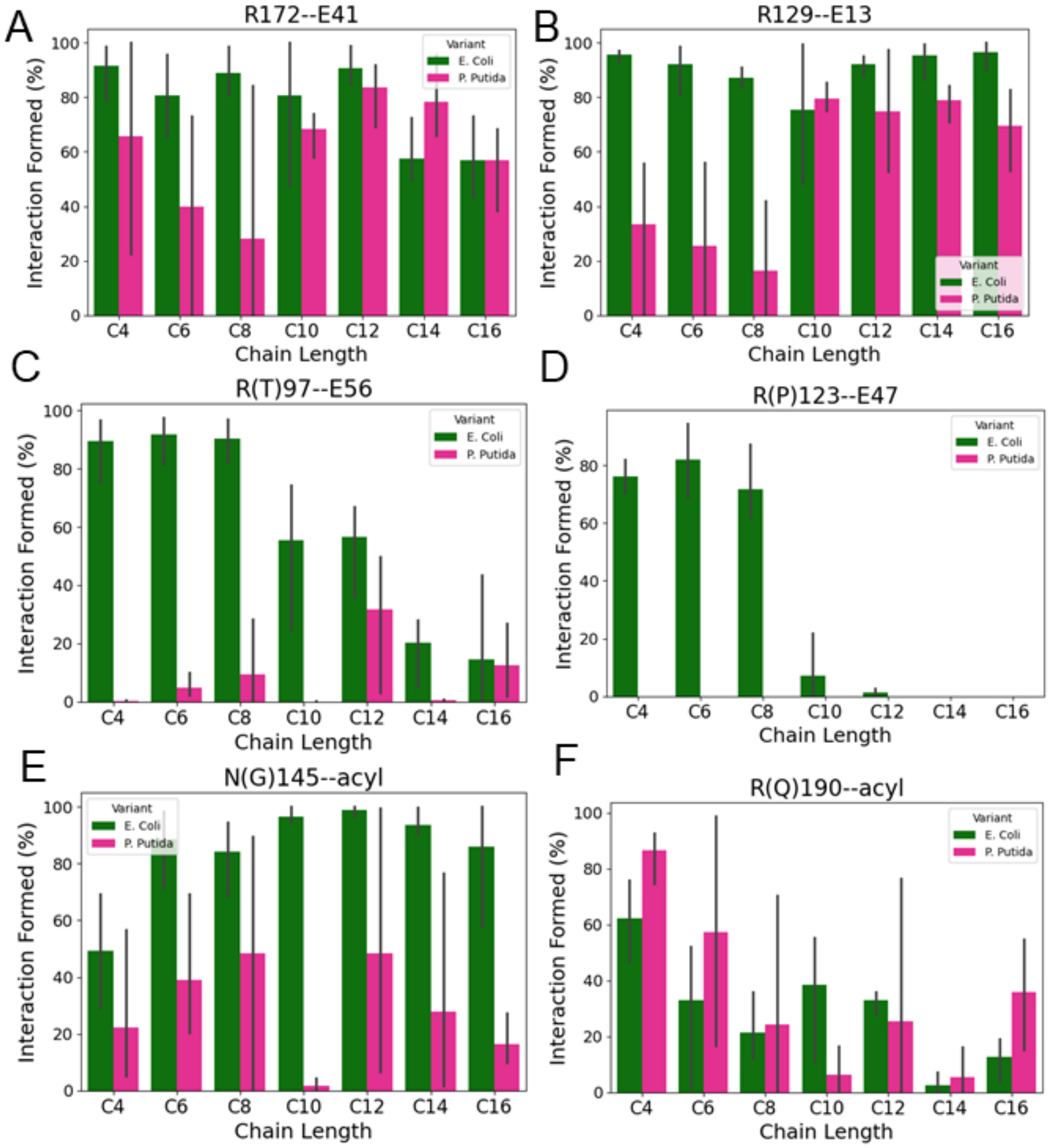

Figure S16: Comparison of FabG-substrate interactions in *Ec*FabG and *Pp*FabG4. (A-F) Plots show the frequency of non-bonded interactions between residues present in both PP_2783 and *Ec*FabG. Numbering corresponds to *Ec*FabG. When the residue identity is different between the two proteins, the residue for PP_2783 appears in parentheses. (A-B) Two salt bridges between glutamate residues on ACP (E41 and E13) and nearby arginines on FabG (R129 and

R172) were previously found to be crucial to ACP binding[19]. In simulations of *Ec*FabG, these residues form stable interactions with ACP for all chain lengths. In simulations of PP_2783, the corresponding interactions are less frequent—and, likely, less stable—for short chain lengths. (C-D) *Ec*FabG forms two additional salt bridges with ACP when bound to short-chain substrates; PP_2783, however, lacks the necessary residues to form these interactions. (E) In *Ec*FabG, N145 forms hydrogen bonds with the Ppant arm for all chain lengths; in PP_2783, the corresponding residue (G147) is incapable of forming such interactions. Although this interaction is not chain length specific, it is one of the few stabilizing interactions that differs between *Ec*FabG and PP_2783. (F) In PP_2783, R190 interacts with the acyl chain more frequently in complex formations that are not catalytically competent (catalytic competency rates less than 50% - Figure 5B—C), such as those involving short-chain substrates. (A—F) We plot the mean for each substrate with n=3 simulations and the error bars represent the standard error on the mean.

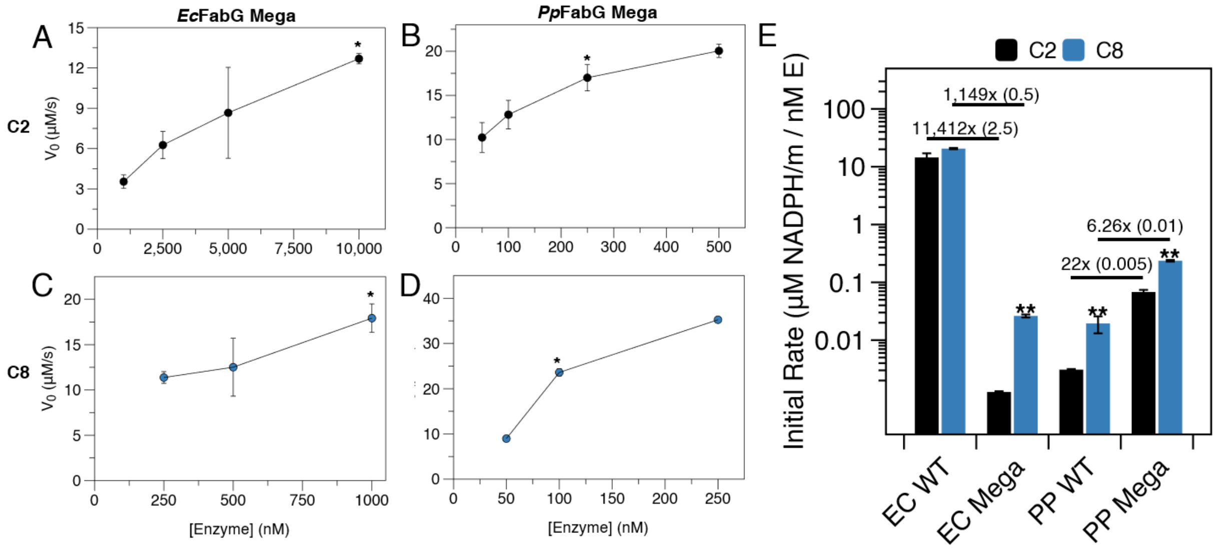

Figure S17. Activity and selectivity of *E. coli* and *P. putida* "Mega" mutants. (A-D) We determine the enzyme concentration at which variants of FabG are rate limiting by reconstituted *E. coli* FASs with (A, C) *Ec*FabG Mega (R97T.R123P.N145G.R190Q.V140A.F183I) and (B, D) *Pp*FabG Mega (T99R.P125R.G147N.Q192R.A142V.I185F) with (A-B) acetyl-CoA or (C-D) octanoyl-CoA. Starred points represent selected rate-limiting concentrations (listed in Table S4) within the linear response range that were used for reconstituted *E.* coli FAS panel E. FAS compositions: various concentrations of FabG, 1 µM *Ec*FabH or 10 µM *Pp*FabH2, 10 µM 'TesA, 10 µM holo-ACP, 1 µM of all other FAS enzymes, 1.3 mM NADPH, 0.5 mM malonyl-CoA, and 0.1 mM acyl-CoA. Measurement time: 2.5 minutes. Data depicts the mean and SE of $n \geq 2$ technical replicates. (E) Normalized initial rates of fatty acid synthesis by reconstituted *E. coli* FASs with variants of *Ec*FabG and *Pp*FabG4 with (black) acetyl-CoA or (blue) octanoyl-CoA. FAS compositions: same as in description above for panels A-D. Measurement time: 2.5 minutes. Data depicts the mean and SE of $n \geq 3$ technical replicates. Independent t-tests between C2 and C8 activity for individual proteins: *, $p<0.05$; **, $p<0.01$.

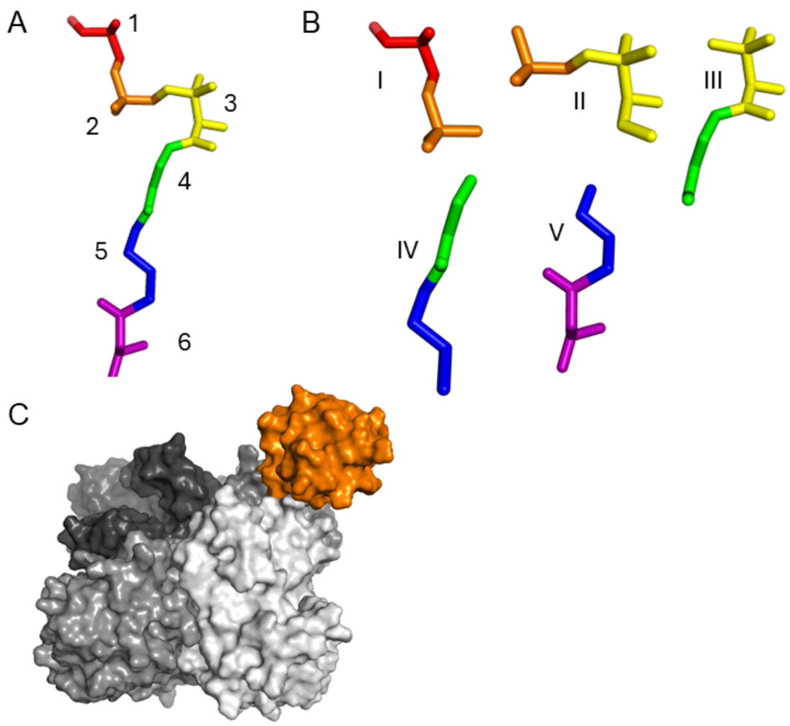

Figure S18. Chain Growth Algorithm. In order to avoid the issues encountered when attempting to perform protein—protein docking with EcFabG and βkACP we implemented this iterative docking protocol. (A) We segment the Ppant and acyl chain into segments which contain 5-8 heavy atoms. The example shown is for a C4 substrate, but the same protocol was applied to the C10 substrate. (B) We created partial chains which each composed of two segments. We generated conformers by varying all rotatable bonds in each one of these partial chains (PC). (C) We took the *Ec*FabG—ACP complex conformations from Rosetta docking as our starting point. We then aligned PC I with the SER residue in the original conformation (segment 1 corresponds to the SER residue). Each new conformation which had the end of PC I pointing towards the catalytic residues (determined by a new closest heavy atom distance less than or equal to the conformation before PC I was added). Then an energy minimization was performed and as long as it converged the conformer proceeds to the next PC. This process continued to add PC II, III, IV, and V.

SI TABLES

Table S1A. FAS-like homologs from *P. putida KT2440.*

Gene #	Putative name	Protein name (this study)	DeepFRI - sequence based molecular function prediction (score)	DeepFRI - sequence based biological process function prediction (score)
PP_0581	3-oxoacyl-[ACP] reductase	FabG 2	oxidoreducatse (0.53)	cellular metabolic process (0.81), organic substance metabolic process (0.85)
PP_1602	3-hydroxyacyl-[acyl-carrier-protein] dehydratase FabZ	FabZ	lyase activity (0.82), transferase (transferring acyl groups) activity (0.82)	lipid biosynthetic process (0.9), cellular lipid metabolic process (0.9)
PP_1852	3-oxoacyl-[ACP] reductase, putative enoyl-[acyl-carrier-protein] reductase (NADPH, B-specific)	FabI 1	Oxidoreductase (0.7)	cellular metabolic process (0.78), organic substance metabolic process (0.78)
PP_1913	malonyl-CoA-ACP transacylase	FabD	no prediction above threshold	cellular metabolic process (0.78), organic substance metabolic process (0.78)
PP_1914	3-oxoacyl-[ACP] reductase	FabG	oxidoreducatse (0.95)	cellular metabolic process (0.9), organic substance metabolic process (0.91)
PP_1915	acyl carrier protein	ACP	no prediction above threshold	organic acid biosynthetic process (0.92), carboxylic acid biosynthetic process (0.92), cellular metabolic process (0.91), organic substance metabolic process (0.89)
PP_1916	3-oxoacyl-[ACP] synthase II; 3-oxo-cis-vaccenoyl-[ACP] synthase	FabF 1	transferase activity (0.58)	cellular metabolic process (0.86), organic substance metabolic process (0.9)
PP_2540	3-oxoacyl-[ACP] reductase	FabG 3	no prediction above threshold	cellular metabolic process (0.75), organic substance metabolic process (0.74)

Table S1B. FAS-like homologs from *P. putida KT2440.*

**Gene #**	**Putative name**	**Protein name (this study)**	**DeepFRI - sequence based molecular function prediction (score)**	**DeepFRI - sequence based biological process function prediction (score)**
PP_2777	putative Acyl carrier protein	ACP	ion binding (0.81), cation binding (0.74), metal ion binding (0.74)	cellular metabolic process (0.53), organic substance metabolic process (0.54)
PP_2778	3-oxoacyl-(Acyl-carrier-protein) synthase II	KS	no prediction above threshold	cellular metabolic process (0.83), organic substance metabolic process (0.85)
PP_2779	putative Beta-ketoacyl synthase	KS	ion binding (0.55)	cellular metabolic process (0.83), organic substance metabolic process (0.82)
PP_2780	3-oxoacyl-(Acyl-carrier-protein) synthase II	KS	no prediction above threshold	cellular metabolic process (0.77), organic substance metabolic process (0.78)
PP_2781	putative Beta-ketoacyl synthase	KS	ion binding (0.56)	cellular metabolic process (0.75), organic substance metabolic process (0.78)
PP_2783	3-oxoacyl-[ACP] reductase	FabG 4	oxidoreductase (0.63)	cellular metabolic process (0.76), organic substance metabolic process (0.74)
PP_3303	3-oxoacyl-[acyl-carrier-protein] synthase II	FabF 2	no prediction above threshold	cellular metabolic process (0.91), organic substance metabolic process (0.9)
PP_4174	3R-3-hydroxydecanoyl acyl carrier protein (ACP) dehydratase	FabA	no prediction above threshold	cellular metabolic process (0.74), organic substance metabolic process (0.73)
PP_4175	3-oxoacyl-[acyl-carrier-protein] synthase 1	FabB	transferase activity (0.76)	cellular metabolic process (0.88), organic substance metabolic process (0.92)
PP_4379	3-oxoacyl-[acyl-carrier-protein] synthase III	FabH 1	no prediction above threshold	cellular metabolic process (0.75), organic substance metabolic process (0.79)
PP_4545	beta-ketodecanoyl-[acyl-carrier-protein] synthase	FabH 2	transferase activity (0.69)	cellular metabolic process (0.72), organic substance metabolic process (0.72)

Table S1C. FAS-like homologs from *P. putida KT2440.*

Gene #	Putative name	Protein name (this study)	DeepFRI - sequence based molecular function prediction (score)	DeepFRI - sequence based biological process function prediction (score)
PP_4635	enoyl-[acyl-carrier protein] reductase / trans-2-enoyl-CoA reductase (NAD+)	FabV	no prediction above threshold	cellular metabolic process (0.87), organic substance metabolic process (0.9)

Table S2A. Plasmids.

**Name**	**Plasmid Base**	**AntR***	**ORI**	**Prom.***	**Gene**	**Ind.***	**Source**
pET16b FabD	pET16b	Cb	pBR322	P_{T7}	*E. coli fabD*	IPTG	(Ruppe et. al., 2020)[20]
pET28a FabA	pET28A	Kan	pBR322	P_{T7}	*E. coli fabA*	IPTG	(Ruppe et. al., 2020)
pET16b FabH	pET16b	Cb	pBR322	P_{T7}	*E. coli fabH*	IPTG	(Ruppe et. al., 2020)
pET15b FabG	pET15b	Cb	pBR322	P_{T7}	*E. coli fabG*	IPTG	(Ruppe et. al., 2020)
pET28a FabZ dimer	pET28A	Kan	pBR322	P_{T7}	*E. coli fabZ*	IPTG	(Ruppe et. al., 2020)
pET16b FabI	pET16b	Cb	pBR322	P_{T7}	*E. coli fabI*	IPTG	(Ruppe et. al., 2020)
pET28a FabB	pET28A	Kan	pBR322	P_{T7}	*E. coli fabB*	IPTG	(Ruppe et. al., 2020)
pET16b FabF	pET16b	Cb	pBR322	P_{T7}	*E. coli fabF*	IPTG	(Ruppe et. al., 2020)
pET28a TesA	pET28A	Kan	pBR322	P_{T7}	*E. coli 'tesA*	IPTG	This study
pET22b ACP	pET22b	Cb	pBR322	P_{T7}	*E. coli acpP*	IPTG	Gift from Michael D. Burkart
pET15b FabG mutants	pET15b	Cb	pBR322	P_{T7}	*E. coli fabG mutants*	IPTG	This study
pET28a FabG mutants	pET28A	Kan	pBR322	P_{T7}	*P. putida FabG mutants*	IPTG	This study
pet28a-PP_0581	pet28a	Kan	pBR322	T7	PP_0581	IPTG	This study
pet28a-PP_1852	pet28a	Kan	pBR322	T7	PP_1852	IPTG	This study
pet28a-PP_1914	pet28a	Kan	pBR322	T7	PP_1914	IPTG	This study
pet28a-PP_2540	pet28a	Kan	pBR322	T7	PP_2540	IPTG	This study

*AntR = Antibiotic Resistance. Prom. = promoter. Ind. = Inducer

Table S2B. Plasmids.

Name	Plasmid Base	AntR *	ORI	Prom. *	Gene	Ind. *	Source
pet28a-PP_2783	pet28a	Kan	pBR322	T7	PP_2783	IPTG	This study
pet28a-PP_4379 FabH-1	pet28a	Kan	pBR322	T7	PP_4379 FabH-1	IPTG	This study
pet28a-PP_4545 FabH-2	pet28a	Kan	pBR322	T7	PP_4545 FabH-2	IPTG	This study
pet28a-PP_1915 ACP	pet28a	Kan	pBR322	T7	PP_1915 ACP	IPTG	This study
pet28A-PP_2777	pet28a	Kan	pBR322	T7	PP_2777	IPTG	This study

*AntR = Antibiotic Resistance. Prom. = promoter. Ind. = Inducer

Table S3A. Primers and DNA Fragments.

Name	Piece	Forward Primer	Reverse Primer
pET28a TesA	1	AAACACGGAAACCGAA GACCATTCATG	AATAACGTGTCCGCTGC CATGAATTCGGAT
	2	ATCCGAATTCATGGCA GCGGACACGTTATT	AGCTTGTCGACGGAGCT CTTATGAGTCATGATTTA
	3	TAAATCATGACTCATA AGAGCTCCGTCGACAA GCT	CATGAATGGTCTTCGGTT TCCGTGTTT
pET15b FabG.R97T	1	AAACACGGAAACCGAA GACCATTCATG	TTCATCTTTCATggtCATT AACAGGTT
	2	AACCTGTTAATGaccAT GAAAGATGAA	CATGAATGGTCTTCGGTT TCCGTGTTT
pET15b FabG.R123P	1	AAACACGGAAACCGAA GACCATTCATG	TTTCATCATAGCcggCATT ACCGCTTT
	2	AAAGCGGTAATGccgGC TATGATGAAA	CATGAATGGTCTTCGGTT TCCGTGTTT
pET15b FabG.R97T. R123P	1	AAACACGGAAACCGAA GACCATTCATG	TTCATCTTTCATggtCATT AACAGGTT
	2	AACCTGTTAATGaccAT GAAAGATGAA	TTTCATCATAGCcggCATT ACCGCTTT
	3	AAAGCGGTAATGccgGC TATGATGAAA	CATGAATGGTCTTCGGTT TCCGTGTTT
pET15b FabG.R97T. R123P.N145G	1	TTTCGCCACCTCTGACT TGAGCGTCGATTTTTGT GATGCTCGTCA	TAGTTGGCCTGACCGCC accTCCCATGGTACCAA
	2	TTGGTACCATGGGAggt GGCGGTCAGGCCAACT A	TGACGAGCATCACAAAA ATCGACGCTCAAGTCAG AGGTGGCGAAA
pET15b FabG.R97T. R123P.R190Q	1	TTTCGCCACCTCTGACT TGAGCGTCGATTTTTGT GATGCTCGTCA	TGGTCATCGCTCAGCGC ctgTGTCATGTCCGTTTC AATAAA
	2	TTTATTGAAACGGACA TGACAcagGCGCTGAGC GATGACCA	TGACGAGCATCACAAAA ATCGACGCTCAAGTCAG AGGTGGCGAAA

Table S3B. Primers and DNA Fragments.

Name	Piece	Forward Primer	Reverse Primer
pET15b FabG.R97T.R123P.N145G.R190Q	1	TTTCGCCACCTCTGACTTGAGCGTCGATTTTTGTGATGCTCGTCA	TAGTTGGCCTGACCGCCaccTCCCATGGTACCAA
	2	TTTATTGAAACGGACATGACAcagGCGCTGAGCGATGACCA	TGACGAGCATCACAAAAATCGACGCTCAAGTCAGAGGTGGCGAAA
	3	TTGGTACCATGGGAggtGGCGGTCAGGCCAACTA	TGGTCATCGCTCAGCGCctgTGTCATGTCCGTTTCAATAAA
pET28a FabG.P125R	1	AAACACGGAAACCGAAGACCATTCATG	ATCATGCAgcgCAGCACCT
	2	AGGTGCTGcgcTGCATGAT	CATGAATGGTCTTCGGTTTCCGTGTTT
pET28a FabG.T99R.P125R	1	AAACACGGAAACCGAAGACCATTCATG	TTCAACGATTGacgGGCGAGCAAA
	2	TTTGCTCGCCcgtCAATCGTTGAA	ATCATGCAgcgCAGCACCT
	3	AGGTGCTGcgcTGCATGAT	CATGAATGGTCTTCGGTTTCCGTGTTT
pET28a FabG.T99R.P125R.G147N	1	AAACACGGAAACCGAAGACCATTCATG	TTGCTCTGGCCCTTgttGGGCTTTT
	2	AAAAGCCCaacAAGGGCCAGAGCAA	CATGAATGGTCTTCGGTTTCCGTGTTT
pET28a FabG.T99R.P125R.Q192R	1	AAACACGGAAACCGAAGACCATTCATG	ACCAGGGCacgGCTCATAT
	2	ATATGAGCcgtGCCCTGGT	CATGAATGGTCTTCGGTTTCCGTGTTT
pET28a FabG.T99R.P125R.G147N.Q192R	1	AAACACGGAAACCGAAGACCATTCATG	TTGCTCTGGCCCTTgttGGGCTTTT
	2	AAAAGCCCaacAAGGGCCAGAGCAA	ACCAGGGCacgGCTCATAT
	3	ATATGAGCcgtGCCCTGGT	CATGAATGGTCTTCGGTTTCCGTGTTT
pet28a-PP_0581	1	ATATAGCTAGCTTGCCTTTCGGGAGACCC	ACATAGAATTCTCAAGCCCCCATCAGTGC

Table S3C. Primers and DNA Fragments.

Name	Piece	Forward Primer	Reverse Primer
pet28a-PP_1852	1	ATATAGCTAGCATGTCCAAGCAACTTACACTCG	ACATAGAATTCTCAGGCTGCAAACCCAC
pet28a-PP_1914	1	ATATAGCTAGCATGAGCCTGCAAGGTAAAGTTG	ACATAGAATTCTTACATGTACATCCCGCCGT
pet28a-PP_2540	1	ATATAGCTAGCATGGATCTAGGCATCACCGG	ACATAGAATTCTCAGTAGGTTCCGGGGTAC
pet28a-PP_2783	1	ATATAGCTAGCATGACTCAGAAAATAGCTGTCGTG	ACATAGAATTCTCATGGCATTTTCAATCCGCC
pet28a-PP_4379<br>FabH-1	1	CATATGGCTAGCATGATTGGCATCAAAAGCATTGCG	GAGCTCGAATTCTCAGTCGCGGTACAGGATGG
	2	CATCCTGTACCGCGACTGAGAATTCGAGCTCCGTCGACAAG	GTAATTGTCCTTTTAACAGCGATCGCGTATTTCG
	3	CGAAATACGCGATCGCTGTTAAAAGGACAATTAC	CACGCCCTCGCTCAAGCCTTC
	4	GAAGGCTTGAGCGAGGGCGTG	CAATGCTTTTGATGCCAATCATGCTAGCCATATGGCTGCCG
pet28a-PP_4545<br>FabH-2	1	CATATGGCTAGCATGTTTCCAGCCAGCGCC	GAGCTCGAATTCTCAGCGCTTGCGCAGAATC
	2	GATTCTGCGCAAGCGCTGAGAATTCGAGCTCCGTCGACAAG	GTAATTGTCCTTTTAACAGCGATCGCGTATTTCG
	3	CGAAATACGCGATCGCTGTTAAAAGGACAATTAC	CACGCCCTCGCTCAAGCCTTC
	4	GAAGGCTTGAGCGAGGGCGTG	CACAGGGCGCTGGCTGGAAACATGCTAGCCATATGGCTGCCG

Table S3D. Primers and DNA Fragments.

Name	Piece	Forward Primer	Reverse Primer
pet28a-PP_1915 ACP	1	CATATGGCTAGCATGAGCACCATCGAAGAACGC	GAGCTCGAATTCTTAGGCCTGGTGGGCTTTG
	2	CAAAGCCCACCAGGCCTAAGAATTCGAGCTCCGTCGACAAG	GTAATTGTCCTTTTAACAGCGATCGCGTATTTCG
	3	CGAAATACGCGATCGCTGTTAAAAGGACAATTAC	CACGCCCTCGCTCAAGCCTTC
	4	GAAGGCTTGAGCGAGGGCGTG	GTTCTTCGATGGTGCTCATGCTAGCCATATGGCTGCCG
pet28A-PP_2777	1	CGGCAGCCATATGAATAATCCGTTGGAATTGGACAGCG	GAGCTCGAATTCCTACGCCGAAGCCTTCAACTGG
	2	ATCCGAATTCATGGCAG CGGACACGTTATT	AGCTTGTCGACGGAGCTCTTATGAGTCATGATTTA
	3	TAAATCATGACTCATAA GAGCTCCGTCGACAAGC T	CATGAATGGTCTTCGGTTTCCGTGTTT
pET15b FabG.V140A	1	AAACACGGAAACCGAAGACCATTCATG	ATTTCCCATGGTACCAGCCACAGAA
	2	TTCTGTGGCTGGTACCATGGGAAAT	CATGAATGGTCTTCGGTTTCCGTGTTT
pET15b FabG.F183I	1	AAACACGGAAACCGAAGACCATTCATG	TCCGTTTCAATAATGCCCGGAGCAA
	2	TTGCTCCGGGCATTATTGAAACGGA	CATGAATGGTCTTCGGTTTCCGTGTTT
pET15b FabG.V140A.F183I	1	AAACACGGAAACCGAAGACCATTCATG	ATTTCCCATGGTACCAGCCACAGAA
	2	TTCTGTGGCTGGTACCATGGGAAAT	TCCGTTTCAATAATGCCCGGAGCAA
	3	TTGCTCCGGGCATTATTGAAACGGA	CATGAATGGTCTTCGGTTTCCGTGTTT

Table S3E. Primers and DNA Fragments.

pET28a FabG.A142V	1	AAACACGGAAACCGAA GACCATTCATG	GCTTTTGCGCGACCACCG AACTGA
	2	TCAGTTCGGTGGTCGC GCAAAAGC	CATGAATGGTCTTCGGTT TCCGTGTTT
pET28a FabG.I185F	1	AAACACGGAAACCGAA GACCATTCATG	ATATCGGTGCTGACGAA GCCGGGC
	2	GCCCGGCTTCGTCAGC ACCGATAT	CATGAATGGTCTTCGGTT TCCGTGTTT
pET28a FabG.A142V .I185F	1	AAACACGGAAACCGAA GACCATTCATG	GCTTTTGCGCGACCACCG AACTGA
	2	TCAGTTCGGTGGTCGC GCAAAAGC	ATATCGGTGCTGACGAA GCCGGGC
	3	GCCCGGCTTCGTCAGC ACCGATAT	CATGAATGGTCTTCGGTT TCCGTGTTT
pET15B FabG.Mega (R97T.R123P.N145G .R190Q.V140A .F183I)	All	See primers from "pET15b FabG.V140A.F183I" and "pET15b FabG.R97T. R123P.N145G.R190Q"	See primers from "pET15b FabG.V140A.F183I" and "pET15b FabG.R97T. R123P.N145G.R190Q"
pET28A FabG.Mega (T99R.P125R.G147N .Q192R.A142V .I185F)	All	See primers from "pET28a FabG.A142V.I185F" and "pET28a FabG.T99R. P125R.G147N.Q192R"	See primers from "pET28a FabG.A142V.I185F" and "pET28a FabG.T99R. P125R.G147N.Q192R"

Table S4A. Selected rate limiting FabG concentrations.

Protein	Assay Substrate	Rate-Limiting FabG Concentration (nM)
E. coli FabG WT	Acetyl-CoA (C2)	2.5
	Octanoyl-CoA (C8)	2.5
E. coli FabG.V140A	Acetyl-CoA (C2)	100
	Octanoyl-CoA (C8)	10
E. coli FabG.F183I	Acetyl-CoA (C2)	100
	Octanoyl-CoA (C8)	10
E. coli FabG.V140A.F183I	Acetyl-CoA (C2)	500
	Octanoyl-CoA (C8)	20
E. coli FabG.R97T	Acetyl-CoA (C2)	5
	Octanoyl-CoA (C8)	5
E. coli FabG.R123P	Acetyl-CoA (C2)	5
	Octanoyl-CoA (C8)	5
E. coli FabG.R97T.R123P	Acetyl-CoA (C2)	5
	Octanoyl-CoA (C8)	5
E. coli FabG.R97T.R123P.N145G	Acetyl-CoA (C2)	40
	Octanoyl-CoA (C8)	20
E. coli FabG.R97T.R123P.R190Q	Acetyl-CoA (C2)	5
	Octanoyl-CoA (C8)	5
E. coli FabG.R97T.R123P.N145G.R190Q	Acetyl-CoA (C2)	40
	Octanoyl-CoA (C8)	20
E. coli FabG.R97T.R123P.V140A.N145G. F183I.R190Q	Acetyl-CoA (C2)	10000
	Octanoyl-CoA (C8)	1000
P. putida FabG WT	Acetyl-CoA (C2)	5000
	Octanoyl-CoA (C8)	500
P. putida FabG.A142V	Acetyl-CoA (C2)	1000
	Octanoyl-CoA (C8)	500
P. putida FabG.I185F	Acetyl-CoA (C2)	1000
	Octanoyl-CoA (C8)	500
P. putida FabG.A142V.I185F	Acetyl-CoA (C2)	500
	Octanoyl-CoA (C8)	500
P. putida FabG.P125R	Acetyl-CoA (C2)	1000
	Octanoyl-CoA (C8)	1000
P. putida FabG.T99R.P125R	Acetyl-CoA (C2)	250
	Octanoyl-CoA (C8)	250

Table S4B. Selected rate limiting FabG concentrations.

P. putida FabG.T99R.P125R.G147N	Acetyl-CoA (C2)	100
	Octanoyl-CoA (C8)	100
P. putida FabG.T99R.P125R.Q192R	Acetyl-CoA (C2)	250
	Octanoyl-CoA (C8)	250
P. putida FabG.T99R.P125R.G147N.Q192R	Acetyl-CoA (C2)	100
	Octanoyl-CoA (C8)	50
P. putida FabG.T99R.P125R.A142V.G147N. I185F.Q192R	Acetyl-CoA (C2)	250
	Octanoyl-CoA (C8)	100

Table S5. Proposed KS initiation mechanism in the absence of an acyl-CoA substrate

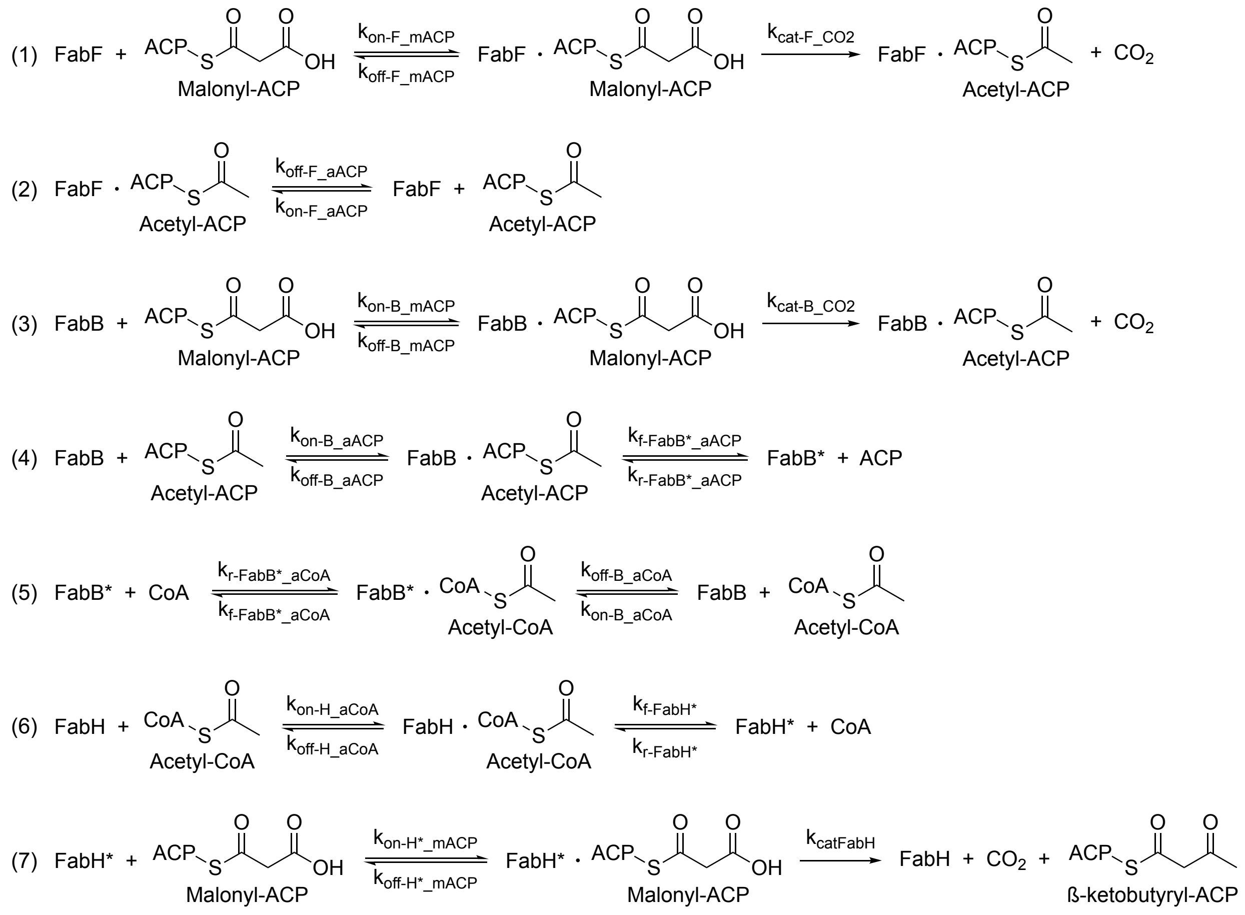

Table S6. Data collection and refinement statistics for X-ray crystallography

	*Pp*FabG4 (TBD)
**Data collection**	
Space group	P 21 21 21
Cell dimensions	
a, *b*, *c* (Å)	59.72, 114.18, 144.53
α, β, γ (°)	90, 90, 90
Resolution (Å)[a]	49.69 – 1.91 (1.95 - 1.91)
R_{merge}[a]	0.234 (2.187)
R_{pim}[a]	0.080 (0.745)
<I/σ(I)>[a]	8.5 (1.1)
$CC_{1/2}$[a]	0.996 (0.322)
Completeness (%)[a]	99.7 (98.4)
Multiplicity[a]	9.1 (9.2)
**Refinement**	
Resolution (Å)	1.91
No. reflections	77212
R_{work} / R_{free}	0.1720 / 0.1986
No. atoms	
Protein A, B, C, D	1746, 1811, 1751, 1757
Water	588
B-factors[b]	
Protein A, B, C, D	29, 28, 33, 28
R.m.s. deviations	
Bond lengths (Å)	0.010
Bond angles (°)	1.217

[a]Values in parentheses are for highest-resolution shell. We examined one crystal.
[b]Values correspond to mean *B*-factors for the indicated atoms.

Table S7. The accompanying Excel file provides raw data, separated by figures, for all experiments described in this work.

SI REFERENCES

[1] X. Xiao, X. Yu, C. Khosla, *Biochemistry* **2013**, *52*, 8304–8312.
[2] K. Mains, J. Peoples, J. M. Fox, *Metab Eng* **2022**, *69*, 209–220.
[3] P. Emsley, K. Cowtan, *Acta Crystallogr D Biol Crystallogr* **2004**, *60*, 2126–2132.
[4] R. P. Joosten, F. Long, G. N. Murshudov, A. Perrakis, *IUCrJ* **2014**, *1*, 213–220.
[5] W. Cao, B. Demeler, *Biophys J* **2005**, *89*, 1589–1602.
[6] J. J. Gray, S. Moughon, C. Wang, O. Schueler-Furman, B. Kuhlman, C. A. Rohl, D. Baker, *J Mol Biol* **2003**, *331*, 281–299.
[7] P. C. D. Hawkins, A. G. Skillman, G. L. Warren, B. A. Ellingson, M. T. Stahl, *J Chem Inf Model* **2010**, *50*, 572–584.
[8] J. A. Maier, C. Martinez, K. Kasavajhala, L. Wickstrom, K. E. Hauser, C. Simmerling, *J Chem Theory Comput* **2015**, *11*, 3696–3713.
[9] W. L. Jorgensen, J. Chandrasekhar, J. D. Madura, R. W. Impey, M. L. Klein, *J Chem Phys* **1983**, *79*, 926–935.
[10] M. Bernetti, G. Bussi, *J Chem Phys* **2020**, *153*, , DOI 10.1063/5.0020514.
[11] G. Bussi, D. Donadio, M. Parrinello, *J Chem Phys* **2007**, *126*, , DOI 10.1063/1.2408420.
[12] Lindahl, Abraham, Hess, van der Spoel, **2022**, Zenodo preprint.
[13] R. T. McGibbon, K. A. Beauchamp, M. P. Harrigan, C. Klein, J. M. Swails, C. X. Hernández, C. R. Schwantes, L. P. Wang, T. J. Lane, V. S. Pande, *Biophys J* **2015**, *109*, 1528–1532.
[14] Q. Guo, C. Zhong, H. Dong, J. E. Cronan, H. Wang, *Journal of Biological Chemistry* **2024**, *300*, , DOI 10.1016/j.jbc.2023.105600.
[15] A. Hurley, M. G. Chevrette, N. Rosario-Meléndez, J. Handelsman, *mBio* **2022**, *13*, e02486-21.
[16] G. L. Lozano, H. B. Park, J. I. Bravo, E. A. Armstrong, J. M. Denu, E. V. Stabb, N. A. Broderick, J. M. Crawford, J. Handelsman, *Appl Environ Microbiol* **2019**, *85*, , DOI 10.1128/AEM.03058-18.
[17] J. Jumper, R. Evans, A. Pritzel, T. Green, M. Figurnov, O. Ronneberger, K. Tunyasuvunakool, R. Bates, A. Žídek, A. Potapenko, A. Bridgland, C. Meyer, S. A. A. Kohl, A. J. Ballard, A. Cowie, B. Romera-Paredes, S. Nikolov, R. Jain, J. Adler, T. Back, S. Petersen, D. Reiman, E. Clancy, M. Zielinski, M. Steinegger, M. Pacholska, T. Berghammer, S. Bodenstein, D. Silver, O. Vinyals, A. W. Senior, K. Kavukcuoglu, P. Kohli, D. Hassabis, *Nature* **2021**, *596*, 583–589.
[18] J. Abramson, J. Adler, J. Dunger, R. Evans, T. Green, A. Pritzel, O. Ronneberger, L. Willmore, A. J. Ballard, J. Bambrick, S. W. Bodenstein, D. A. Evans, C. C. Hung, M. O'Neill, D. Reiman, K. Tunyasuvunakool, Z. Wu, A. Žemgulytė, E. Arvaniti, C. Beattie, O. Bertolli, A. Bridgland, A. Cherepanov, M. Congreve, A. I. Cowen-Rivers, A. Cowie, M. Figurnov, F. B. Fuchs, H. Gladman, R. Jain, Y. A. Khan, C. M. R. Low, K. Perlin, A. Potapenko, P. Savy, S. Singh, A. Stecula, A. Thillaisundaram, C. Tong, S. Yakneen, E. D. Zhong, M. Zielinski, A. Žídek, V. Bapst, P. Kohli, M. Jaderberg, D. Hassabis, J. M. Jumper, *Nature* **2024**, *630*, 493–500.
[19] Y. M. Zhang, B. Wu, J. Zheng, C. O. Rock, *Journal of Biological Chemistry* **2003**, *278*, 52935–52943.
[20] A. Ruppe, K. Mains, J. M. Fox, *Proc Natl Acad Sci U S A* **2020**, *117*, , DOI 10.1073/pnas.2013924117.
